# PCI-DB: a novel primary tissue immunopeptidome database to guide next-generation peptide-based immunotherapy development

**DOI:** 10.1136/jitc-2024-011366

**Published:** 2025-04-15

**Authors:** Steffen Lemke, Marissa L Dubbelaar, Patrick Zimmermann, Jens Bauer, Annika Nelde, Naomi Hoenisch Gravel, Jonas Scheid, Marcel Wacker, Susanne Jung, Anna Dengler, Yacine Maringer, Hans-Georg Rammensee, Cecile Gouttefangeas, Sven Fillinger, Tatjana Bilich, Jonas S Heitmann, Sven Nahnsen, Juliane S Walz

**Affiliations:** 1 Department of Peptide-based Immunotherapy, Institute of Immunology, University and University Hospital Tübingen, Tübingen, BW, Germany; 2 Cluster of Excellence iFIT (EXC2180) “Image-Guided and Functionally Instructed Tumor Therapies”, University of Tübingen, Tübingen, BW, Germany; 3 Quantitative Biology Center (QBiC), University of Tübingen, Tübingen, BW, Germany; 4 Department of Computer Science, University of Tübingen, Tübingen, BW, Germany; 5 Institute for Bioinformatics and Medical Informatics (IBMI), University of Tübingen, Tübingen, BW, Germany; 6 German Cancer Consortium (DKTK) and German Cancer Research Center (DKFZ), partner site Tübingen, Tübingen, BW, Germany; 7 Clinical Collaboration Unit Translational Immunology, German Cancer Consortium (DKTK), Department of Internal Medicine, University Hospital Tübingen, Tübingen, BW, Germany; 8 Institute of Immunology, University of Tübingen, Tübingen, BW, Germany; 9 M3 Research Center, University Hospital of Tübingen, Tübingen, BW, Germany

**Keywords:** Immunotherapy, Vaccine, Human leukocyte antigen - HLA

## Abstract

**Background:**

Various cancer immunotherapies rely on the T cell-mediated recognition of peptide antigens presented on human leukocyte antigens (HLA). However, the identification and selection of naturally presented peptide targets for the development of personalized as well as off-the-shelf immunotherapy approaches remain challenging.

**Methods:**

Over 10,000 raw mass spectrometry (MS) files from over 3,000 tissue samples were analyzed, summing to approximately seven terabytes of data. The raw MS data were processed using the standardized and open-source nf-core pipelines MHCquant2 and epitopeprediction, providing a uniform procedure for data handling. A global false discovery rate was applied to minimize false-positive identifications.

**Results:**

Here, we introduce the open-access Peptides for Cancer Immunotherapy Database (PCI-DB, https://pci-db.org/), a comprehensive resource of immunopeptidome data originating from various malignant and benign primary tissues that provides the research community with a convenient tool to facilitate the identification of peptide targets for immunotherapy development. The PCI-DB includes >6.6 million HLA class I and >3.4 million HLA class II peptides from over 40 tissue types and cancer entities. First application of the database provided insights into the representation of cancer-testis antigens across malignant and benign tissues, enabling the identification and characterization of cross-tumor entity and entity-specific tumor-associated antigens (TAAs) as well as naturally presented neoepitopes from frequent cancer mutations. Further, we used the PCI-DB to design personalized peptide vaccines for two patients suffering from metastatic cancer. In a retrospective analysis, PCI-DB enabled the composition of both a multi-peptide vaccine comprising non-mutated, highly frequent TAAs matching the immunopeptidome of the individual patient’s tumor and a neoepitope-based vaccine matching the mutational profile of a patient with cancer. Both vaccine approaches induced potent and long-lasting T-cell responses, accompanied by long-term survival of these patients with advanced cancer.

**Conclusion:**

The PCI-DB provides a highly versatile tool to broaden the understanding of cancer-related antigen presentation and, ultimately, supports the development of novel immunotherapies.

**WHAT IS ALREADY KNOWN ON THIS TOPIC**

**WHAT THIS STUDY ADDS**

- The Peptides for Cancer Immunotherapy Database (PCI-DB) includes over 6.6 million HLA class I and over 3.4 million HLA class II peptides from over 40 tissue types and cancer entities, enabling the identification and characterization of cross-tumor entity and entity-specific tumor-associated antigens. This study provides insights into the representation of cancer-testis antigens across malignant and benign tissues. Further, it shows the retrospective design of personalized peptide vaccines for patients with advanced cancer, which induced potent and long-lasting T-cell responses and accompanied long-term survival of the patients.

**HOW THIS STUDY MIGHT AFFECT RESEARCH, PRACTICE OR POLICY**

- The PCI-DB represents a highly versatile and dynamic immunopeptidome database that broadens the understanding of cancer-related antigen presentation and supports the development of future novel immunotherapies.

## Background

 T-cell recognition of peptides presented via human leukocyte antigens (HLA) on the cell surface is crucial for immune surveillance of malignant diseases.^1 2^ Thus, HLA peptide antigens play a pivotal role in the induction of therapeutic T-cell responses against cancer, particularly for adoptive T-cell transfer and cancer vaccines.^3^,^6^ The development of such immunotherapeutic approaches requires suitable peptide targets specific to the tumor and not presented on benign tissue to minimize the risk of on-target off-tumor toxicities.^7 8^ One source of such cancer-specific targets is neoepitopes, which arise from DNA mutations that alter amino acid sequences and are subsequently presented on the cell surface via HLA molecules. The mutational burden and HLA-presented neoepitopes have been shown to correlate strongly with responses to immune checkpoint blockade.^9 10^ However, intratumoral heterogeneity of somatic mutations and the limited number of mutations that are presented as HLA-restricted neoepitopes on the tumor cell,^911^,^14^ currently hamper the broad applicability of mutation-derived neoepitopes, in particular in low-mutational burden cancer entities.^15^ Tumor-associated antigens (TAAs), arising from canonical proteins and exclusively presented on HLA molecules of tumor cells, represent a further source of antigens. These peptides result from dysregulated expression of genes or differential protein processing in cancer.^16 17^ Immunotherapeutic approaches using both neoepitopes and TAAs have shown the first glimmers of success in recent years.^5 6 18 19^ However, for a broad and effective clinical application of immunotherapies in large patient populations, tools for efficiently identifying and selecting naturally presented tumor antigens that go beyond current prediction algorithms are required.

Identification and characterization of naturally presented HLA ligands on the cell surface are enabled through mass spectrometry (MS)-guided immunopeptidomics, which has experienced advancements in instrumentation, computational speed, and the development of more sensitive analysis pipelines and software tools in recent years.^20^,^23^ This increased the number of detectable HLA peptides in samples to thousands of peptides, thereby enabling large-scale characterization of the unique immunopeptidome of various diseases and benign tissues.^1324^,^26^ Within the last decade, numerous groups have adopted methodologies to investigate the immunopeptidomes of various malignant diseases. This led to the foundation of the Human Immunopeptidome Project (HIPP), which aims to foster technological developments, standardization, and data sharing.^27 28^ A central tenet of the HIPP is data sharing, which is realized through databases such as PRoteomics IDEntifications (PRIDE),^29^ enabling data reuse and increasing the data basis. Existing public immunopeptidome databases, which have extensively reanalyzed and integrated these public data, provide comprehensive resources for peptide searching, data mining, and developing new algorithms.^26 30 31^ However, high-confidence immunopeptidome-based identification of tumor antigens requires comparisons using large, well-annotated datasets from both primary malignant and benign tissue. Furthermore, it is central to perform cancer entity-specific and tissue-specific querying and filtering of the immunopeptidome to identify disease-specific patterns, which in turn allow the identification of novel peptide targets.

Here, we present the open-access database “Peptides for Cancer Immunotherapy Database” (PCI-DB), a comprehensive collection of immunopeptidome data originating from various malignant and benign primary tissues that provides the research community with a user-friendly tool for the selection of peptide targets for immunotherapy development. The first application of the database offers (1) insights into cross-tumor entity and entity-specific TAAs, (2) the representation of cancer-testis antigens (CTA) across malignant and benign tissues, (3) naturally presented neoepitopes from frequent cancer mutations, and (4) first evidence for the feasibility and efficacy of the PCI-DB guided design and application of personalized peptide vaccines for patients with advanced cancer.

## Results

### PCI-DB provides an extensive source of HLA peptides from malignant and benign origin

**Figure 1:**
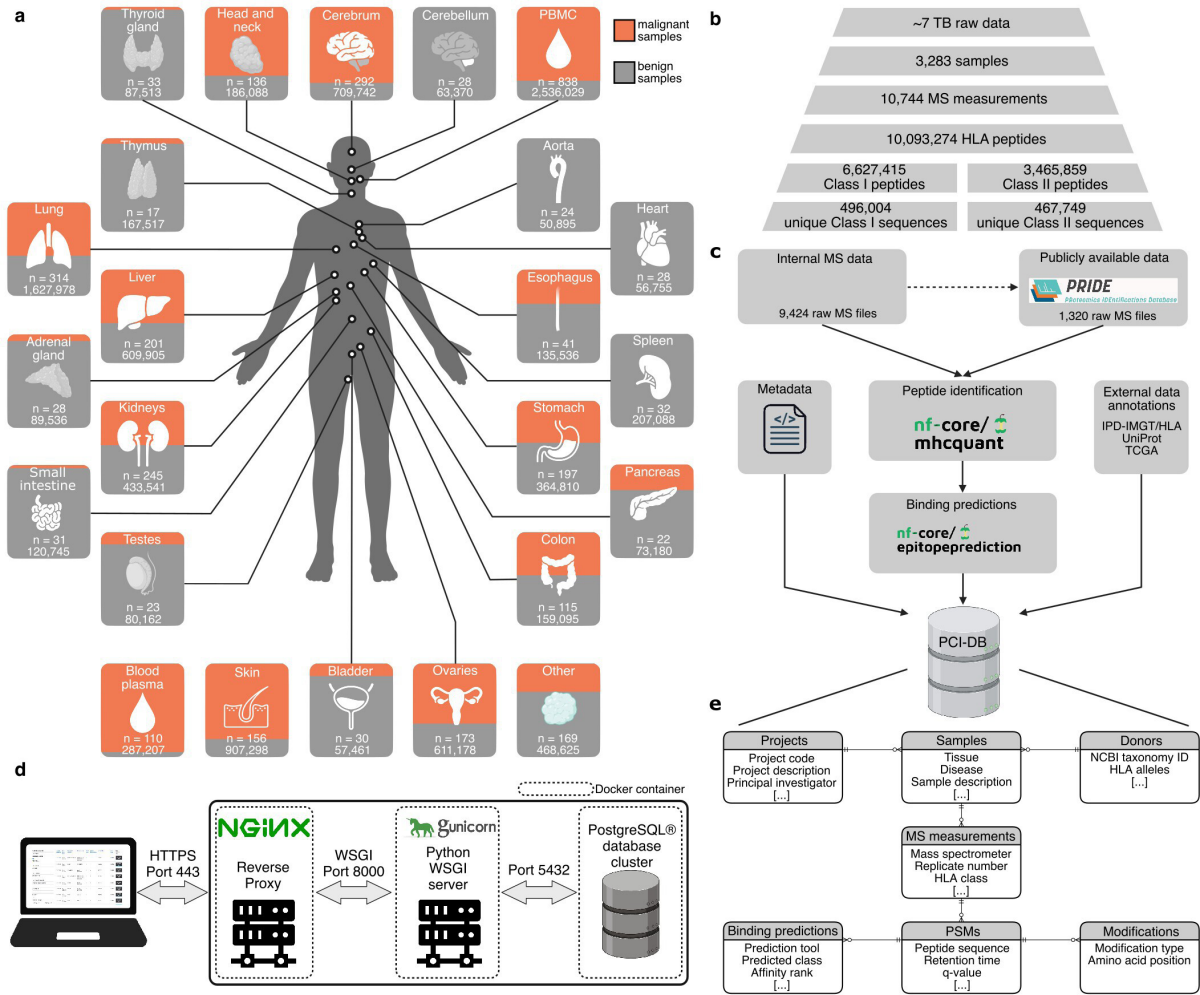
Summary of data content of the Peptides for Cancer Immunotherapy Database (PCI-DB). (A**a**) Overview of tissue samples included in the PCI-DB. The ratio of malignant (orange) to benign (gray) samples is represented by the proportion of the box coloring. The total number of samples (n) across malignant and benign is indicated in each box, and the total number of peptides is displayed at the bottom of each box. (B) Quantitative summary of raw data and the identified HLA peptides. (C) Data processing workflow of the PCI-DB, which incorporates raw MS data, external database information, and metadata. (D) Schematic overview of the database server back-end structure. (E) Entity-relationship diagram of the database scheme in which the data and metadata of PCI-DB are stored. HLA, human leukocyte antigen; HTTPS, Hypertext Transfer Protocol Secure; ID, identifier; MS, mass spectrometry; NCBI, National Center for Biotechnology Information; PBMC, peripheral blood mononuclear cells; PRIDE, PRoteomics IDEntifications; PSM, peptide spectrum match; TB, terabyte; TCGA, The Cancer Genome Atlas; WSGI, Web Server Gateway Interface.^71^

The PCI-DB provides a comprehensive collection of human HLA class I and II peptide data consisting of both novel and publicly available human data from various malignant and benign primary tissue samples (figure 1a). The database facilitates exploration of the immunopeptidome and supports the research community to identify target peptides or proteins for immunotherapeutic application. The PCI-DB is made available through a web interface (https://pci-db.org), which provides access to the peptide data and allows for querying and filtering options. The database builds on 1,320 raw MS files from publicly available data stored in PRIDE, as well as 9,424 in-house MS files, from a total of 3,283 samples, summing to approximately 7 terabytes of data. The raw MS data were processed using the standardized and open-source nf-core pipelines MHCquant2^23 32^ and epitopeprediction^33^ providing a uniform procedure for data handling. This resulted in 6,627,415 and 3,465,859 HLA class I and II peptides, respectively, with 496,004 unique HLA class I peptide sequences and 467,749 unique HLA class II peptide sequences included in the PCI-DB (figure 1b). Analysis with the isoform database revealed an increase of 2,567 unique HLA class I peptides, representing a 0.5% increase; however, for HLA class II, a decrease of 236 unique peptide sequences, or 0.1%, was observed (online supplemental figure 1). The PCI-DB comprises three selectable database versions depending on the researcher’s needs: (1) UP000005640 reference proteome, (2) UP000005640 with protein isoforms, and (3) UP000005640 with the 1,000 most frequent The Cancer Genome Atlas (TCGA) missense mutations. Each database version was generated using a global peptide false discovery rate (FDR) approach for HLA class I and II samples to minimize the accumulation of false-positive identifications. The HLA peptide data were combined with metadata from the samples and stored in a relational database, ensuring data integrity and avoiding redundancy (figure 1c,e). Validation tables are used to control the vocabulary (*e.g.,* disease, tissue) (online supplemental figure 2). The web application for the PCI-DB was designed with easy deployability in mind. Each component of the PCI-DB web application, the PostgreSQL cluster, the back-end server implemented in Django, and the Nginx, which serves as a reverse proxy, are containerized in separate Docker containers (figure 1d). Although the web application allows querying for peptide sequences, it additionally provides multiple interactive visualizations to facilitate the exploration and a manual visualization option for each MS/MS spectrum.

**Figure 2:**
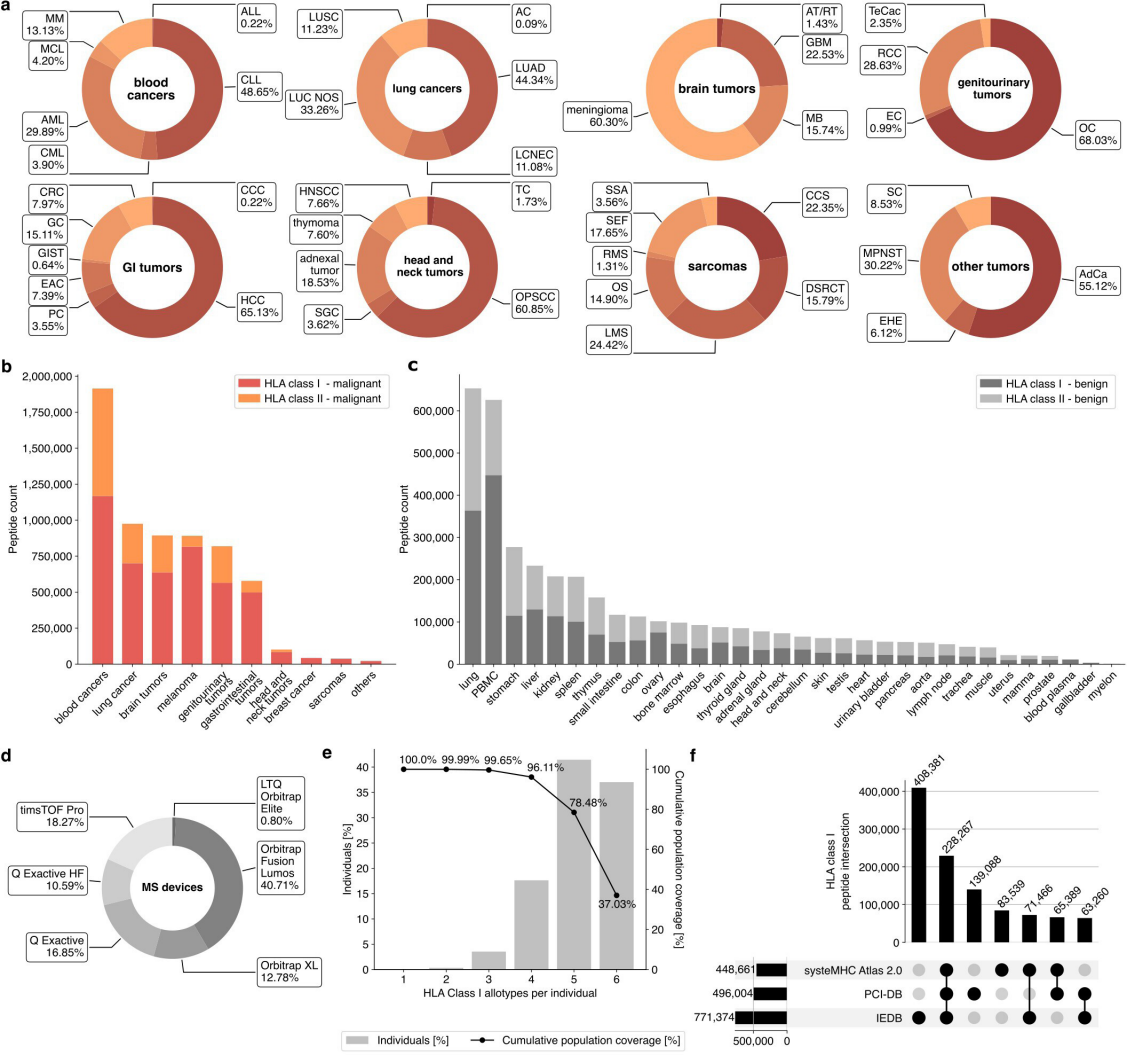
PCI-DB composition of HLA peptides from benign and malignant tissues and benchmarking. (**a**A) Cancer entities are classified into different disease groups based on anatomical location with the number of HLA peptides for each disease group (B) and the relative proportion of peptides for each cancer entity.(C) Overview of HLA peptides identified in different benign tissues. (D) Percentages of peptides derived from different MS devices. (E) Global population coverage of the HLA class I alleles contained in the database. Gray bars display the population percentage with the number of alleles in the database (left y-axis). The black line and dots show the cumulative population coverage in percent (right y-axis). (F) Overlap analysis of unique peptides shared between PCI-DB, IEDB,^34^ and systeMHC Atlas v2.0.^31^ AC, atypical carcinoid of the lung; AdCa, adrenal cancer; ALL, acute lymphocytic leukemia; AML, acute myeloid leukemia; AT/RT, atypical teratoid rhabdoid tumor; CCC, cholangiocellular carcinoma; CCS, clear cell sarcoma; CLL, chronic lymphocytic leukemia; CML, chronic myeloid leukemia; CRC, colorectal cancer; DSRCT, desmoplastic small round cell tumors; EAC, esophageal carcinoma; EC, endometrial cancer; EHE, epithelioid hemangioendothelioma; GBM, glioblastoma; GC, gastric carcinoma; GI, gastrointestinal; GIST, gastrointestinal stromal tumor; HCC, hepatocellular carcinoma; HLA, human leukocyte antigen; HNSCC, head and neck squamous cell carcinoma; IEDB, Immune Epitope Database; LCNEC, large cell neuroendocrine carcinoma; LMS, leiomyosarcoma; LUAD, lung adenocarcinoma; LUC NOS, lung cancer not otherwise specified; LUSC, lung squamous cell carcinoma; MB, medulloblastoma; MCL, mantle cell lymphoma; MM, multiple myeloma; MPNST, malignant peripheral nerve sheath tumor; MS, mass spectrometry; OC, ovarian carcinoma; OPSCC, oropharyngeal squamous cell carcinoma; OS, osteosarcoma; PBMC, peripheral blood mononuclear cell; PC, pancreatic carcinoma; PCI-DB, Peptides for Cancer Immunotherapy Database; RCC, renal cell carcinoma; RMS, rhabdomyosarcoma; SC, sarcomatoid carcinoma; SEF, sclerosing epithelioid fibrosarcoma; SGC, salivary gland cancer; SSA, synovial sarcoma; TC, thyroid cancer; TeCac, testicular cancer.

The PCI-DB comprises immunopeptidome data from 45 different solid and hematological tumor entities. Cancers related to blood (n=614) and lungs (n=166) represent the largest disease groups, with 1,167,884 HLA class I and 746,572 HLA class II peptides identified for blood cancer and 700,344 HLA class I and 274,636 HLA class II peptides for lung cancers. 48.7% of blood cancer-derived peptides were detected in samples of patients with chronic lymphocytic leukemia (CLL) (n=148) and 29.9% of patients with acute myeloid leukemia (n=237). For lung cancer, lung adenocarcinoma (n=52) represents the largest cancer entity, with 44.3%. Further, meningiomas (n=160) account for 60.3% of brain tumors, comprising a total of 893,873 HLA class I and II peptides, while ovarian carcinomas (OvCa) account for 68.0% (n=113) of genitourinary tumors, comprising 795,971 HLA class I and II peptides (figure 2a,b).

In addition, the PCI-DB contains immunopeptidome data from 32 different benign tissue types as a reference source to identify HLA-presented antigen targets for cancer immunotherapy that show malignant-exclusive presentation. The highest yields of peptides were found for lung tissue (n=134) and peripheral blood mononuclear cells (PBMC, n=143), with more than 652,998 and 625,759 HLA peptides, respectively (figure 2c). The samples were measured on a variety of MS devices, with 68.95% using Orbitrap technology (figure 2d). Pairwise distance analysis of all HLA class I and II immunopeptidomes shows no clear clustering by MS device (online supplemental figure 3a,b). For HLA class II, samples measured on Q Exactive and Q Exactive HF devices show a trend for local grouping, which, however, correlates with the HB-145 and HB-298 antibodies used during the immunoprecipitation step (online supplemental figure 3c). The different MS devices from which immunopeptidome data were integrated into this study also show differences in median peptide yield ranging from 6,463 (timsTOF Pro) to 1,088 peptides per sample (Orbitrap XL, online supplemental figure 3d). The HLA allotypes, for which there are predicted HLA-binding peptides in the PCI-DB, provide 99.7% coverage for at least three alleles for HLA class I and 99.6% coverage for at least five alleles for HLA class II of the global population (figure 2e, online supplemental figure 4a). This indicates that the PCI-DB holds peptides relevant to the vast majority of the world’s population, making it a valuable resource for immunotherapy approaches. Benchmarking the PCI-DB with other large-scale public repositories for immunopeptidome data, the PCI-DB provides a unique set of 139,088 HLA class I and 230,398 HLA class II peptides, which cannot be obtained from the HLA database systeMHC Atlas v2.0^31^ and the Immune Epitope Database (IEDB)^34^ (figure 2f, online supplemental figure 4b).

### The PCI-DB delineates TAAs across various cancer entities

**Figure 3:**
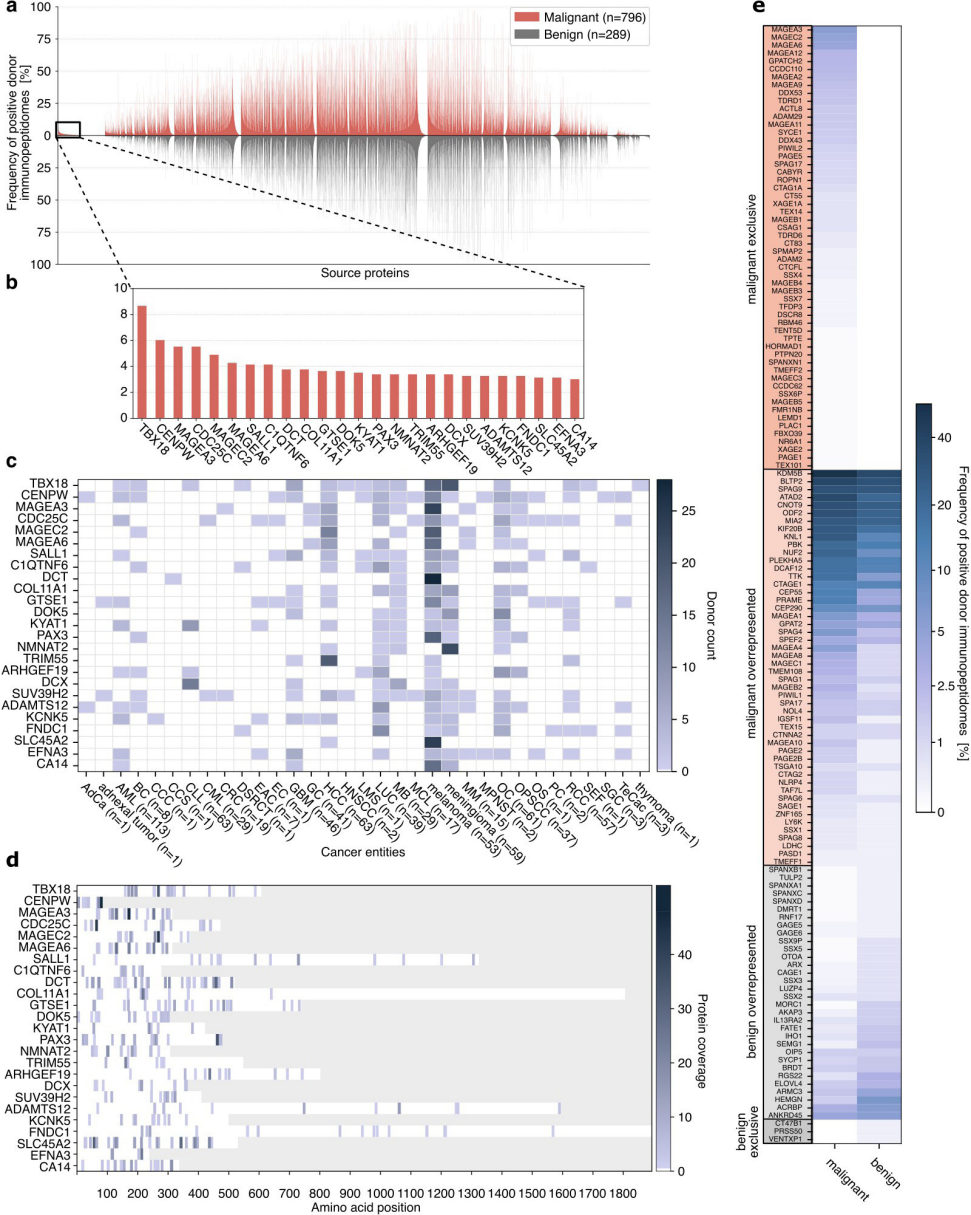
Characterization of tumor-associated proteins in HLA class I immunopeptidomes across all samples of the PCI-DB. (A) Comparative profiling of HLA class I source proteins, comparing malignant and benign immunopeptidomes. The frequency of positive immunopeptidomes is shown on the y-axis, the source proteins on the x-axis. Proteins with a frequency<0.5% were omitted. The box on the left and (B), its magnified view, highlight the 25 most frequently identified malignant-exclusive proteins. (C) Frequency of HLA class I peptides derived from the 25 most abundant malignant-exclusive proteins in patients with cancer. (D) Protein coverage of malignant-exclusive proteins. The coverage of each amino acid position describes how often an amino acid was detected as part of a peptide. (E) Analysis of CTAs represented within the HLA class I immunopeptidomes.^65^ Percentage of donors with identification of peptides originating from CTAs, grouped into four categories: malignant-exclusive, benign-exclusive, and malignant-over-represented/benign-over-represented. AdCa, adrenal cancer; AML, acute myeloid leukemia; BC, breast cancer; CCC, cholangiocellular carcinoma; CCS, clear cell sarcoma; CLL, chronic lymphocytic leukemia; CML, chronic myeloid leukemia; CRC, colorectal cancer; CTA, cancer-testis antigen; DSRCT, desmoplastic small round cell tumor; EAC, esophageal cancer; EC, endometrial cancer; GBM, glioblastoma; GC, gastric cancer; HCC, hepatocellular carcinoma; HLA, human leukocyte antigen; HNSCC, head and neck squamous cell carcinoma; LMS, leiomyosarcoma; LUC, lung cancer; MB, medulloblastoma; MCL, mantel cell lymphoma; MM, multiple myeloma; MPNST, malignant peripheral nerve sheath tumor; OC, ovarian carcinoma; OPSCC, oropharyngeal squamous cell carcinoma; OS, osteosarcoma; PC, pancreatic cancer; PCI-DB, Peptides for Cancer Immunotherapy Database; RCC, renal cell carcinoma; SEF, sclerosing epithelioid fibrosarcoma; SGC, salivary gland cancer; TeCac, testicular cancer.

Comparative profiling of HLA class I and II source proteins was performed using all malignant and benign donor samples of the PCI-DB to identify tumor-exclusive proteins shared across different cancer entities (figure 3a, online supplemental figure 5a). The analysis revealed 1,398 HLA class I and 3,259 HLA class II tumor-exclusive proteins, respectively (online supplemental figure 6a,b). The most frequently identified malignant-exclusive HLA class I and II source proteins are the T-box transcription factor (TBX18, n=69) and the probable phospholipid-transporting ATPase IM (ATP8B4, n=40) found in 8.7% and 7.1% of malignant donor immunopeptidomes, respectively (figure 3b, online supplemental figure 5b). Notably, the CTAs MAGEA3, MAGEC2, and MAGEA6, currently evaluated for T cell-based immunotherapy approaches, rank among the most frequent malignant-exclusive proteins (figure 3b).^35 36^

Cancer entities were analyzed for the presentation of peptides from the most abundant tumor-exclusive proteins. The highest number of immunopeptidomes comprising HLA class I peptides from these tumor-exclusive source proteins can be found in melanoma (n=53), and meningioma (n=59), which also rank among the most prominent cancer entities in our dataset (figure 3c). For HLA class II, a comparable pattern was observed with the CLL cohort (n=72), representing the largest patient cohort of the PCI-DB, comprising the most HLA class II peptides derived from the tumor-exclusive proteins (online supplemental figure 5c). Of note, HLA peptides from tumor-exclusive source proteins primarily cluster in small hotspot areas of a protein (figure 3d, online supplemental figure 5d). Furthermore, CTAs, whose expression is typically restricted to gametogenic tissues or tumors,^37^ were analyzed as source proteins for tumor-associated HLA peptide targets. A total of 5,984 and 530 CTA-derived HLA class I and II peptides were identified in the PCI-DB. 56 and 31 CTAs were identified as malignant-exclusive source proteins in HLA class I and II immunopeptidomes, with MAGEA3 being the most frequently identified for HLA class I and BLTP2 for HLA class II. Additionally, 50 CTAs were identified to be over-represented in HLA class I and 13 in HLA class II (figure 3e, online supplemental figure 5e).

Similar to the comparative profiling for HLA source proteins, profiling at the HLA peptide level was conducted. Overlap analysis revealed 225,932 HLA class I peptides and 163,053 HLA class II peptides to be exclusively detected on tumor tissue (online supplemental figure 6c,d). For HLA class I, the 25 most abundant tumor-exclusive peptides were detected in up to 6.4% of all malignant donor immunopeptidomes (online supplemental figure 7a,b). Five of these highly abundant HLA class I peptides exhibit a length of ≥14 amino acids, with three originating from Vimentin, a protein in intermediate filaments. These three Vimentin-derived peptides were identified 61 times within the same project and are not predicted HLA binders (online supplemental figure 7c,d), pointing to a possible project-specific contamination as the median NetMHCpan rank of all Vimentin-derived peptides is below the binding threshold, indicating an HLA restriction (online supplemental figure 8a). The length distribution shows that longer Vimentin-derived HLA class I peptides (≥11 amino acids) are detected relatively more frequently compared with all identified peptides. However, the general distribution of peptide lengths remains similar, with a typical peak of 9-mers for HLA class I peptides (online supplemental figure 8b). Furthermore, gene expression analysis of the tissues most frequently included in the PCI-DB shows that Vimentin is highly expressed across different tissues, which might contribute to its abundant detection in immunopeptidomics studies (online supplemental figure 8c-e). To assess the quality of the other most highly abundant peptides, the binding affinity to corresponding donor HLA allotypes was evaluated. 11/25 of the most abundant HLA class I tumor-exclusive peptides show a median affinity rank of less than two, qualifying as high-affinity binders (online supplemental figure 7d). These HLA-binding peptides were identified in various tumor entities within the PCI-DB and are not exclusive to one cancer entity or project. For HLA class II, the 25 most abundant tumor-exclusive peptides were detected in up to 11.3% of all malignant donor immunopeptidomes, of which 18 were frequently identified in CLL samples (online supplemental figure 9a-d). In contrast to HLA class I, only 5/25 of these most abundant tumor-exclusive HLA class II peptides show a median affinity rank of less than two, qualifying as high-affinity binders (online supplemental figure 9e).

### PCI-DB provides tumor entity-specific peptides and naturally presented neoepitopes from frequent mutations as targets for immunotherapy

**Figure 4:**
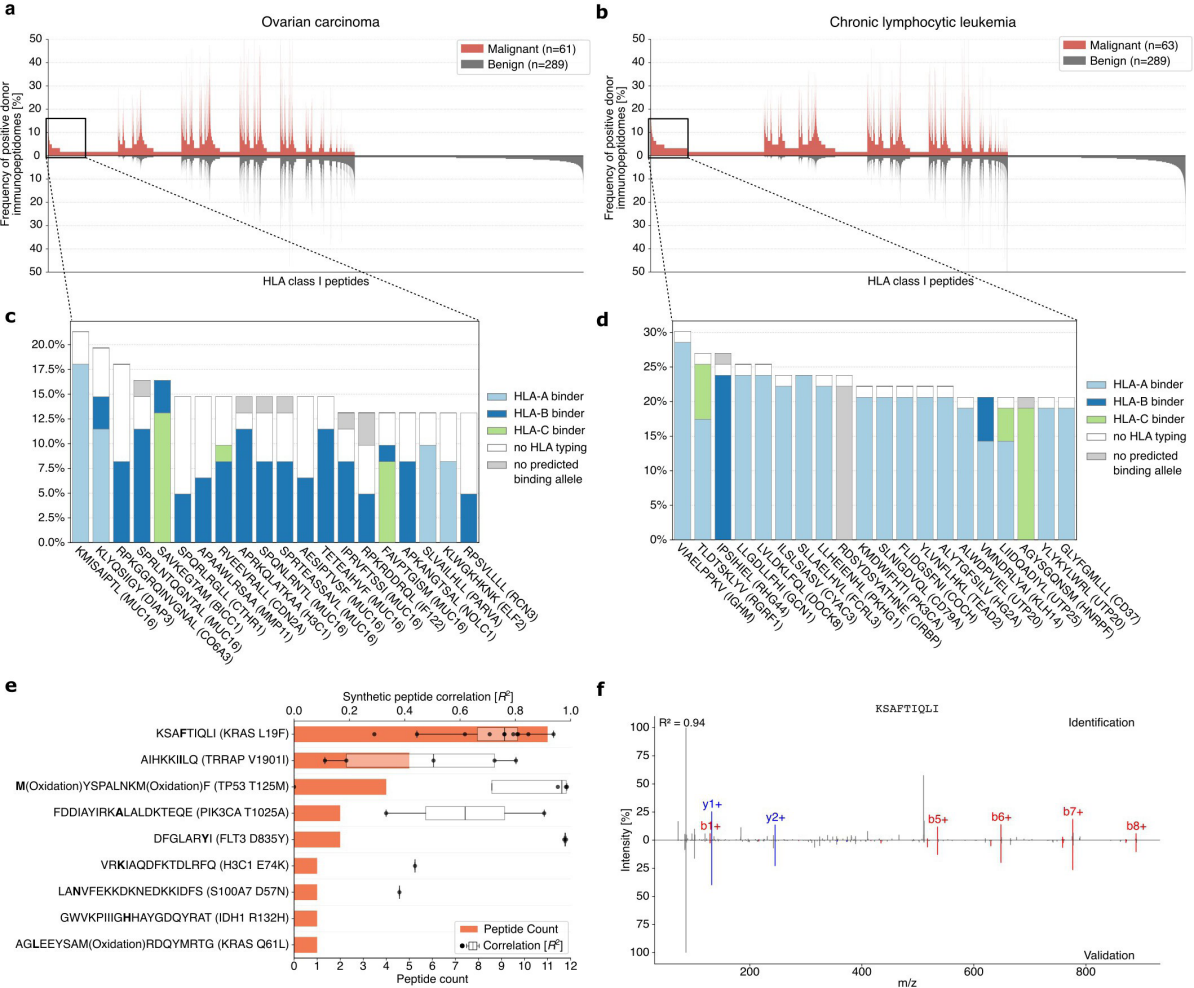
Peptide target identification for cancer immunotherapy. (A,B) Comparative profiling of HLA class I immunopeptidomes of OvCa (A) and CLL (B) samples compared with all benign data. Frequency of positive donor immunopeptidomes (y-axis) for each peptide (x-axis). Peptides with a frequency<0.5% were omitted. (C,D) Magnification of the most abundant OvCa-exclusive peptides (C) and CLL-exclusive peptides (D). Each sequence is annotated with its respective source protein. Colors indicate to which HLA allele the peptides are predicted by NetMHCpan4.1. (E) Frequency of peptide detection across different samples with R² comparing MS-identified neoepitope candidates to corresponding synthetic peptides. (F) Fragment spectrum validation of the neoepitope candidate KSAFTIQLI (KRAS L19F). The experimentally eluted peptide (identification) is shown above the x-axis, the corresponding synthetic peptide below the x-axis (validation). Matching b-ions and y-ions are displayed in red and blue, respectively. CLL, chronic lymphocytic leukemia; HLA, human leukocyte antigen; OvCa, ovarian carcinoma.

As the frequency of tumor-exclusive peptides across all tumor entities only reaches 6.4%, we investigated individual tumor entities, specifically OvCa (n=61) and CLL (n=63), to identify high-frequent peptide candidates for cancer immunotherapy development. Similar to the entity-spanning peptide analysis, comparative profiling of HLA class I peptides against all benign samples contained in the PCI-DB was performed. For OvCa, tumor-exclusive peptides were identified with frequencies of up to 21% (figure 4c). These abundant OvCa-exclusive peptides are predicted to predominantly bind to HLA-B allotypes, particularly HLA-B*07:02 (online supplemental figure 10a). Of note, 8 of the 20 most abundant tumor-exclusive HLA class I peptides originate from MUC16, which is a high‐molecular‐weight glycoprotein and well-described tumor antigen in OvCa^24 38^ (figure 4a and c). In the CLL cohort, tumor-exclusive peptides were identified with a frequency of up to 30% (figure 4b and d), with HLA-A*02:01 representing the allotype for 17 of the 20 most abundant peptides (online supplemental figure 10b). An HLA allotype-specific search for frequent tumor-exclusive peptides within the OvCa and CLL cohort further revealed HLA-A*02:01 tumor-exclusive peptides with frequencies of up to 41.7%, and 54.5%, respectively (online supplemental figure 11a-d). Similarly, comparative profiling for HLA class II peptides for OvCa (n=49) and CLL (n=58) against all benign samples was performed. Tumor-exclusive peptides for OvCa samples exhibit frequencies of up to 24.5% (online supplemental figure 12a,c). The majority of the 20 most abundant tumor-exclusive HLA class II peptide identifications for OvCa are either no predicted binders or were detected in donor samples without HLA typing. Analysis revealed that 5 of the 20 abundant tumor-exclusive HLA class II peptides originate from HNRNPA2B1, a ribonucleoprotein. In the CLL cohort, tumor-exclusive HLA class II peptides were found with a frequency of up to 48.3% (online supplemental figure 12b,d). Binding prediction for the most frequently found peptides in the CLL cohort could not be conducted due to a lack of HLA typing.

In addition to the PCI-DB-guided identification of novel off-the-shelf tumor-exclusive antigens from non-mutated proteins, the database enables the screening for naturally presented neoepitopes derived from tumor-specific mutations. The inclusion of neoantigen search within the PCI-DB was enabled due to recent improvements in the rescoring methods in MHCquant2 that largely increased the sensitivity in the detection of low-abundant peptides.^32^ A neoantigen search against the 1,000 most frequent missense mutations of the TCGA using all available data in the PCI-DB was conducted. This large-scale neoepitope search revealed nine different neoepitope peptide sequences in 28 overall detections (figure 4e). For validation, the spectra of the identified neoepitope candidates were compared with their corresponding synthetic peptide spectra. The peptide KSAFTIQLI (KRAS L19F) was found in 11 malignant samples, with a median R² of 0.79 across all synthetic peptides comparisons and a highest R² score of 0.94 (figure 4e,f, online supplemental figure 13). In addition, the peptides MYSPALNKMF (TP53 T125M) and DFGLARYI (FLT3 D835Y) show a median R² value over the different identifications of >0.95, indicating the accuracy of their identification.

### PCI-DB enables the design of personalized peptide vaccines that mediate potent immune responses in patients with cancer

**Figure 5:**
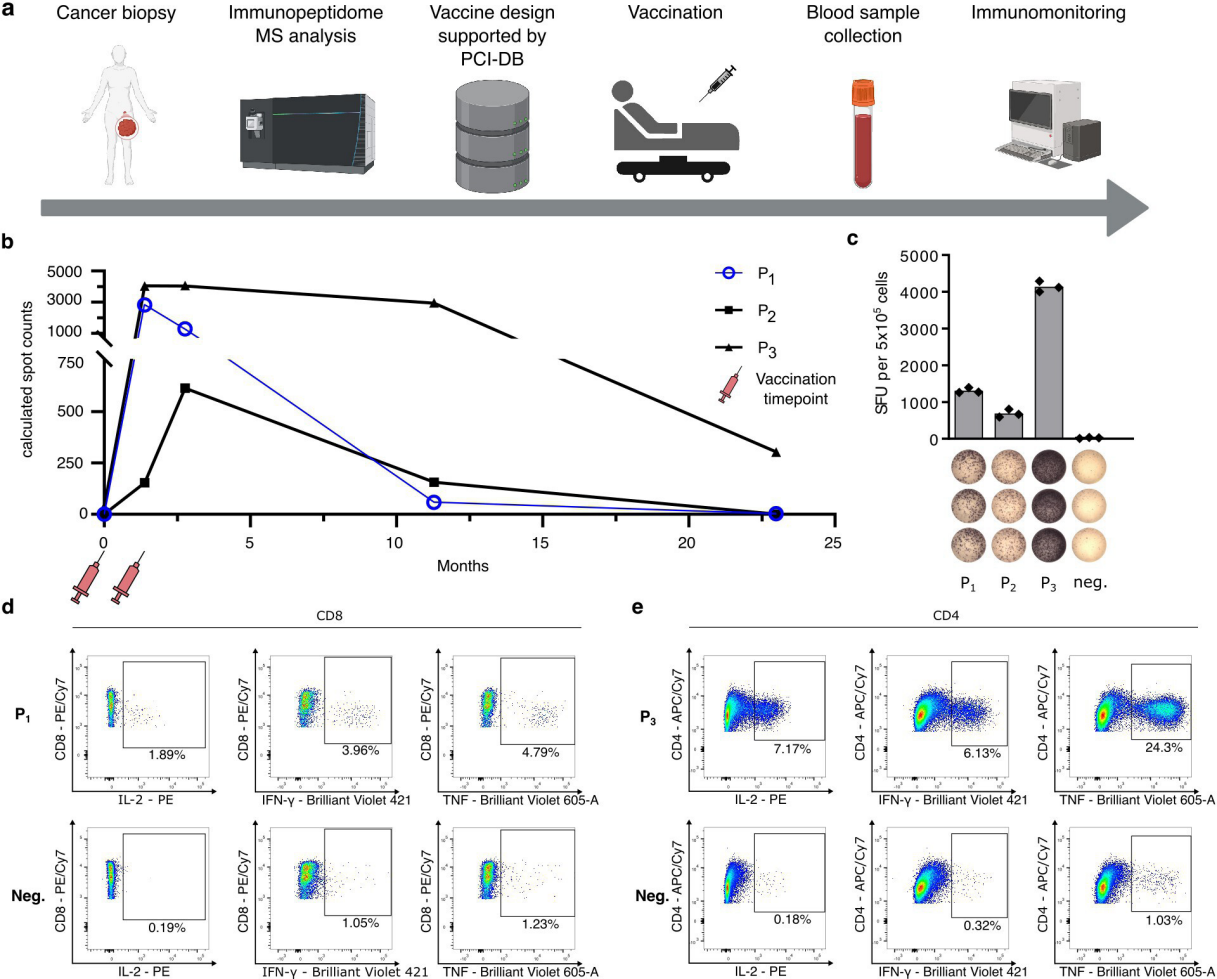
Therapeutic cancer vaccination of a patient with ovarian carcinoma with a PCI-DB-guided personalized multi-peptide vaccine. (A) Schematic overview of personalized vaccine design, application and monitoring. MS-based immunopeptidome analysis was conducted from a tumor biopsy. High-frequency tumor-exclusive target peptides were selected with the support of the PCI-DB. Multi-peptide vaccines were administered twice within 6 weeks. (**B**) Longitudinal analysis of peptide-specific T-cell responses by IFN-γ ELISpot assays. (C) IFN-γ ELISpot assay results 6 weeks after the second vaccination. (D,E) Flow cytometric data of CD8^+^ cells (D) stimulated with P1 and CD4^+^ cells (E) stimulated with P3 and intracellularly stained for the cytokines IL-2, IFN-γ and TNF. ELISpot, Enzyme-Linked ImmunoSpot; IFN-γ, interferon gamma; IL-2, interleukin 2; MS, mass spectrometry; PCI-DB, Peptides for Cancer Immunotherapy Database; SFU, spot forming units, TNF, tumor necrosis factor.^72^

The PCI-DB can be used to create personalized peptide vaccine cocktails comprising naturally presented TAAs arising from non-mutated gene products as well as neoepitopes from somatic mutations. The sizeable number of immunopeptidome datasets from primary malignant tissues across various solid tumors enables the selection of peptides frequently found in tumors. At the same time, the large benign tissue immunopeptidome datasets help to exclude peptides found in these tissues, minimizing the risk of on-target off-tumor toxicities. Within a retrospective analysis, the PCI-DB was evaluated for the design of personalized peptide vaccines for two patients with cancer (OvCa01 and ProCa02) with advanced disease (figure 5a). Patient OvCa01 suffered from metastatic ovarian cancer and was treated with cancer therapies comprising multiple surgeries and three lines of chemotherapy. MS-based immunopeptidome analysis was conducted in technical triplicates from a tumor biopsy of a hepatic metastasis. Comparative profiling with the PCI-DB revealed three peptides from the patient’s immunopeptidome that were never presented in any benign tissue and showed a high representation frequency within the malignant datasets (SPQNLRNTL (P_1_), ALHSHMINK (P_2_), and ALASGTGLFK (P_3_)). The peptide SPQNLRNTL was identified ten times in the PCI-DB, exclusively in other OvCa samples. ALHSHMINK was detected in 52 malignant samples, including six OvCa cases, while ALASGTGLFK was found in 22 malignant samples, 4 of which were OvCa (online supplemental table 1). Vaccine peptides were N-terminally elongated to improve the induction of immune responses by both CD4^+^ and CD8^+^ cells.^39^ A multi-peptide vaccine, including P_1_, P_2_ and P_3_, was applied twice within a 6-week interval and adjuvanted with the toll-like receptor (TLR) 1/2 agonist XS15 (Pam3Cys-GDPKHPKSF) emulsified in Montanide ISA51 VG to stimulate the induction of a strong T-cell response by promoting the activation and maturation of antigen-presenting cells and preventing immediate vaccine peptide degradation.^40^,^42^ A potent T-cell response was detected 6 weeks after the second vaccination as quantified by interferon gamma (IFN-γ) Enzyme-Linked ImmunoSpot (ELISpot) assays. The immune responses to P_1_ and P_2_ persisted for over 10 months, while P_3_ still induced a potent T-cell response at the 23-month follow-up test (figure 5b,c). Immune responses were mediated by multifunctional CD8^+^ (P_1_) and CD4^+^ (P_2_) T cells expressing the cytokines interleukin-2 (IL-2), INF-γ, and tumor necrosis factor (figure 5d,e).

The second patient, ProCa02, had been diagnosed with advanced prostate cancer with pulmonary metastases and had received various therapies comprising surgery, radiotherapy, and antihormonal treatment. At the time of the vaccine design, a fresh frozen tumor biopsy for immunopeptidome analysis was unavailable. Screening of the PCI-DB for naturally presented neoepitopes matching the patient’s tumor mutational profile, obtained from gene panel sequencing of a pulmonary metastasis, identified the peptide FDDIAYIRKALALDKTEQE (P_1_) derived from the PIK3CA T1025A mutation. A P_1_-containing personalized multi-peptide vaccine adjuvanted with XS15 emulsified in Montanide ISA51 VG was applied two times within a 4-week interval and a booster at 2 months and 2 years. A potent P_1_-specific T-cell response, mediated by CD4^+^ T-cells expressing the cytokines IL-2 and INF-γ, was induced on vaccination that persisted until the latest follow-up at month 52 thus far (online supplemental figure 14). Of note, despite their advanced disease both patients OvCa01 and ProCa02 showed long-term survival after vaccination for 4 years and up to now 5 years, respectively, pointing to a positive effect of PCI-DB-based vaccine product to cancer immune surveillance.

In summary, the PCI-DB is a comprehensive database of benign and malignant immunopeptidome data that advances the understanding of cancer-related antigen presentation and facilitates the identification of antigen targets for cancer immunotherapy.

## Discussion

The identification and selection of HLA-presented target antigens is central for the development of cancer immunotherapies, including cancer vaccines and adoptive T-cell transfer, but also recently developed T-cell receptor (TCR)-mimicking chimeric antigen receptor T-cell approaches and TCR-like antibody constructs.^3 5 43 44^ This study presents the PCI-DB, a novel, comprehensive, and customizable immunopeptidome data resource for defining HLA-presented peptide targets for cancer immunotherapy. MS-based immunopeptidomics represents the only unbiased method to identify and characterize naturally presented HLA class I- and HLA class II-restricted peptides on the cell surface.^45 46^ The implementation of immunopeptidomics in selection workflows for therapeutically used tumor antigens is of central importance. Particularly regarding the distorted correlation between gene expression and HLA-restricted antigen presentation, with only a fraction of alterations on genome and transcriptome level resulting in an HLA-presented peptide on the tumor cell surface.^13 15 47 48^ Significant differences in the immunopeptidome and resulting antigen repertoire have been shown between primary tissue and in vitro cultured materials from cell lines.^49^ The PCI-DB, in contrast to other resources like the systeMHC Atlas v2.0 or the IEDB,^26 30 31 34^ focuses on primary malignant and benign human tissue samples to ideally support the design of immunotherapies. The systeMHC Atlas v2.0 provides a more general database for immunopeptidomics data from public sources, integrating a wide range of immunopeptidomics experiments across species. The IEDB represents a large resource that integrates the results of various sources on B cell, T cell, and major histocompatibility complex assays. Compared with the IEDB, the PCI-DB provides more detailed metadata to the corresponding peptides and was generated using standardized data processing. While the IEDB provides a valuable catalog of epitopes, it is not tailored toward immunotherapy design. As each of these databases has a slightly different scope, we are convinced that they all play a valuable part in advancing the field of immunopeptidomic-guided immunotherapy. Comparative immunopeptidome profiling is central for selecting tumor antigens to be applied in immunotherapies. A tumor-exclusive presentation without representation of the respective antigen on benign tissue enables tumor-directed immune targeting while minimizing the risk of on-target off-tumor adverse events. This is particularly relevant for TAAs, including CTAs, which arise from canonical gene products from dysregulated cellular processes in the cancer cell. Beyond comparative profiling of benign and malignant immunopeptidomes for tumor-exclusive presentation, the immense data collection for various tumor entities within the PCI-DB enables the identification of frequently presented TAAs to be applied in off-the-shelf immunotherapeutic approaches.^5 50 51^ To provide high-confidence identification of TAAs within the PCI-DB, standardized data processing was conducted using the highly sensitive MHCquant2 pipeline and a global peptide FDR correction for the accumulation of false positives. As the primary goal of the PCI-DB is to aid the identification and comparison of HLA peptide sequences for immunotherapies, only methionine oxidation and cysteine carbamidomethylation were included in data processing as variable modifications to account for frequently observed technical modifications in immunopeptidomics experiments.^52^ The characterization of other post-translational modifications (PTMs) can enable the identification of previously undescribed HLA peptides and, thereby, broaden the immunopeptidome landscape.^52^ However, including an extensive number of PTMs in the analysis of immunopeptidomics data greatly expands the search space, increasing the likelihood of false-positive hits, which decreases the number of peptides found at a fixed FDR. Identifying frequent tumor-exclusive antigens and CTAs across all tumor entities in the database provides first evidence for using PCI-DB in target discovery. However, in line with previous reports, the frequency of tumor-exclusive peptides across all entities was limited.^53^ This frequency could be significantly increased when focusing on specific tumor entities and HLA allotypes.^5^ Using this approach for CLL and OvCa as use case examples for hematological malignancies and solid tumors, PCI-DB enabled the identification of high-frequent tumor-exclusive peptides as prime targets for cancer immunotherapy development. It has been demonstrated that the choice of the sample preparation method influences the repertoire of HLA peptides.^54^ Additionally, technical aspects such as the MS and incoherent data analysis using different tools can affect the detected immunopeptidome.^55 56^ To this end, the PCI-DB includes immunopeptidome data from different sample preparation protocols and various MS devices to provide a more robust basis for TAA identification across projects. Still, imbalances of datasets from different preparation methods and MS devices might bias antigen selection. Sample similarity analysis revealed no clear technical bias, suggesting technical bias does not mask the biological signal. Additionally, differences in identified peptides have already been reported related to hydrophobicity, where peptides measured on ion mobility separation-based time-of-flight devices had a higher hydrophobicity than those measured on Orbitrap devices.^20^ Differences in sample preparation methods and biases by MS devices are challenging issues to overcome. Since there is continuous scientific development of protocols and mass spectrometers, complete standardization most likely will not be achievable. However, as promoted by HIPP,^28^ the scientific exchange of well-established protocols and methodologies and good documentation support their dissemination. Over time, this and technological advances could allow for a convergence of small sets of standardized workflows, similar to the evolution observed in short-read nucleotide sequencing, where the most accurate and cost-effective methods prevailed.^57^ An example of a project-specific bias is the occurrence of Vimentin-derived peptides, which were found repeatedly in a single project. These peptides were not predicted as HLA binders, which emphasizes the importance of integrating binding prediction in downstream analyses to exclude non-HLA-derived peptides from target selection.

Further, the landscape of the whole benign and malignant immunopeptidome is still not covered, and it is expected that the analysis of new samples of additional tissues and diseases will reveal previously undetected HLA peptides. This implies that future expansions of databases with new datasets will be required. The PCI-DB is designed to enable a continuous implementation of new methods for data processing and novel immunopeptidome data from own and public datasets, making it a flexible tool for future antigen target discovery. One major limitation of the incorporation of publicly available data is the availability of metadata for files and samples. The metadata of the projects from PRIDE that is contained in the PCI-DB was obtained through manual curation from the non-standardized sample overviews and publications. This cumbersome process bears the risk of errors. In recent years, PRIDE has been implementing the standardized metadata format Sample and Data Relationship Format (SDRF) to improve the documentation of the relationship between samples and files^29^, which will further enhance the data quality within the PCI-DB in the future.

In addition to the PCI-DB-guided identification of novel off-the-shelf tumor-exclusive antigens from non-mutated proteins, the database enables the screening for naturally presented neoepitopes derived from tumor-specific mutations. In contrast to previous reports, we implemented a global neoepitope search approach that does not rely on patient-based genomic information^13 58^ but uses curated information about frequent mutations from TCGA.^59^ Multiple naturally presented neoepitope candidates were detected across different cancer entities. Recent improvements in MS sensitivity and advanced processing algorithms, as recently reported for the here-used MHCquant2 pipeline,^32^ will further increase the detection of low-abundant HLA peptides of cancer neoepitopes.^20 22 32 60^ For any claim of biological and therapeutical relevance of these neoepitopes, further studies are required to prove their immunogenicity.

We further provide the first evidence for the feasibility and efficacy of the PCI-DB to guide the design of personalized peptide vaccines. After decades of falling short of potential, therapeutic cancer vaccines have recently entered the spotlight of immunotherapy development.^1^,^5^ This low-side effect immunotherapy approach, which relies on the induction of a tumor-specific T cell-based immune response targeting HLA-presented peptides on the tumor cell surface, holds the potential to mediate long-term immune memory and thus sustainable cancer control. In recent work, we have developed immunopeptidome-guided peptide vaccines adjuvanted with the novel TLR 1/2 agonist XS15 as an adjuvant,^40^ which have proven to induce potent and long-lasting CD4^+^ and CD8^+^ T-cell responses accompanied by signs of clinical efficacy from preclinical models until phase II clinical evaluation.^42 61^ Here, we evaluated the potential of the PCI-DB to design personalized peptide vaccines in a retrospective analysis of two patients suffering from metastatic ovarian cancer and prostate cancer, with and without available immunopeptidome data, respectively. PCI-DB enabled the composition of both a multi-peptide vaccine comprising non-mutated high-frequency ovarian cancer-associated antigens matching the immunopeptidome of the individual patient’s tumor and a neoepitope-based vaccine matching the mutational profile of the patient with prostate cancer. For both approaches, the induction of potent, long-lasting, and multifunctional T-cell responses on therapeutic vaccination was shown. Vaccine-induced immune responses were accompanied by improved long-term survival of these patients with advanced cancer, both significantly exceeding the expected median overall survival time of 33 months and 19 months for metastasized ovarian and prostate cancer, respectively.^62 63^ These data indicate, in line with other cancer vaccines,^18 64^ the potential of PCI-DB-guided vaccination approaches to mediate cancer immune surveillance, with future prospective trials in larger patient cohorts needed to validate these single patient observations.

In conclusion, PCI-DB represents a highly versatile and dynamic web-based immunopeptidome database that broadens the understanding of cancer-related antigen presentation and, ultimately, supports the development of novel immunotherapies.

## Methods

Detailed methods can be found in online supplemental file 3.

### Data acquisition

Raw MS data from primary human tissue samples were obtained from PRIDE^65^ and in-house data generated at the Department of Peptide-based Immunotherapy and stored at the core facility Quantitative Biology Center using the qPortal platform.^66^ Online supplemental data S1 provides a detailed description of the projects used to generate the database.

### Database search

MS data were processed using the nf-core/mhcquant pipeline (release V.2.6.0)(online supplemental file 2).^23 32 67^

### HLA binding prediction

HLA binding prediction was performed using the nf-core/epitopeprediction pipeline.^33^

### Comparison to other HLA databases

All downloadable peptide information of the systeMHC Atlas v2.0 was downloaded from the website (https://systemhc.sjtu.edu.cn/, accessed June 23, 2024).^31^ Peptide information of the IEDB^34^ was acquired from the IEDB Database Export webpage (https://www.iedb.org/database_export_v3.php, accessed June 23, 2024) from the epitope_full_v3.csv file. Only linear peptides of human origin detected using MS were considered.

### Population coverage

The global population coverage of the alleles in the database was computed using the IEDB web tool (http://tools.iedb.org/population/, accessed June 23, 2024).^34^ The input considered only alleles with predicted binders in the PCI-DB.

### CTA analysis

For CTA analysis, identity information was obtained from the CTdatabase (http://www.cta.lncc.br/, accessed on January 19, 2024).^65^ Gene names were mapped to UniProt IDs using the UniProt ID Mapping Tool (https://www.uniprot.org/id-mapping).

### Isolation of HLA ligands

HLA peptides were isolated using previously described immunoaffinity chromatography.^68^ The protocols use the pan-HLA class I-specific monoclonal antibody (mAb) W6/32, the pan-HLA class II-specific mAb Tü−39, and the HLA-DR-specific L243 mAb, all produced in-house.

### Spectrum validation

Spectrum validation of the experimentally eluted peptides was performed by computing the similarity of the spectra with corresponding synthetic peptides measured in a complex matrix. Linear regression was fitted to the matching b and y ions of the MS/MS spectra of the eluted and synthetic peptides to assess the correlation (R^2^).^69^

### Personalized peptide vaccine production and application

Composition, production, and application of the personalized peptide vaccine within a compassionate use program (expanded access) for personalized peptide vaccination under the project ClinicalTrials.gov NCT05014607 was performed as described previously.^4^

### Ampliﬁcation of peptide-speciﬁc T-cells and IFN-γ ELISpot assay

PBMCs isolated from patients with cancer within a compassionate use program were stimulated and analyzed as described previously.^4^

### Intracellular cytokine staining

The functionality of vaccine peptide-specific T cells was analyzed by intracellular cytokine staining, as previously described.^5 39 70^

## Supplementary material

10.1136/jitc-2024-011366 online supplemental file 1

10.1136/jitc-2024-011366 online supplemental file 2

10.1136/jitc-2024-011366 online supplemental figure 1

10.1136/jitc-2024-011366 online supplemental file 3

10.1136/jitc-2024-011366 online supplemental file 4

## Data Availability

Data are available in a public, open access repository.

## References

[1] Ryschich E, Nötzel T, Hinz U (2005). Control of T-cell-mediated immune response by HLA class I in human pancreatic carcinoma. Clin Cancer Res.

[2] Gao Q, Liang W-W, Foltz SM (2018). Driver Fusions and Their Implications in the Development and Treatment of Human Cancers. Cell Rep.

[3] van den Berg JH, Heemskerk B, van Rooij N (2020). Tumor infiltrating lymphocytes (TIL) therapy in metastatic melanoma: boosting of neoantigen-specific T cell reactivity and long-term follow-up. J Immunother Cancer.

[4] Bauer J, Köhler N, Maringer Y (2022). The oncogenic fusion protein DNAJB1-PRKACA can be specifically targeted by peptide-based immunotherapy in fibrolamellar hepatocellular carcinoma. Nat Commun.

[5] Nelde A, Maringer Y, Bilich T (2021). Immunopeptidomics-Guided Warehouse Design for Peptide-Based Immunotherapy in Chronic Lymphocytic Leukemia. Front Immunol.

[6] Wick W, Dietrich P-Y, Kuttruff S (2018). GAPVAC-101: First-in-Human Trial of a Highly Personalized Peptide Vaccination Approach for Patients with Newly Diagnosed Glioblastoma.

[7] Morgan RA, Chinnasamy N, Abate-Daga D (2013). Cancer regression and neurological toxicity following anti-MAGE-A3 TCR gene therapy. J Immunother.

[8] Linette GP, Stadtmauer EA, Maus MV (2013). Cardiovascular toxicity and titin cross-reactivity of affinity-enhanced T cells in myeloma and melanoma. Blood.

[9] van Rooij N, van Buuren MM, Philips D (2013). Tumor exome analysis reveals neoantigen-specific T-cell reactivity in an ipilimumab-responsive melanoma. J Clin Oncol.

[10] Snyder A, Makarov V, Merghoub T (2014). Genetic basis for clinical response to CTLA-4 blockade in melanoma. N Engl J Med.

[11] Yadav M, Jhunjhunwala S, Phung QT (2014). Predicting immunogenic tumour mutations by combining mass spectrometry and exome sequencing. Nature New Biol.

[12] Freudenmann LK, Marcu A, Stevanović S (2018). Mapping the tumour human leukocyte antigen (HLA) ligandome by mass spectrometry. Immunology.

[13] Bassani-Sternberg M, Bräunlein E, Klar R (2016). Direct identification of clinically relevant neoepitopes presented on native human melanoma tissue by mass spectrometry. Nat Commun.

[14] Löffler MW, Chandran PA, Laske K (2016). Personalized peptide vaccine-induced immune response associated with long-term survival of a metastatic cholangiocarcinoma patient. J Hepatol.

[15] Bilich T, Nelde A, Bichmann L (2019). The HLA ligandome landscape of chronic myeloid leukemia delineates novel T-cell epitopes for immunotherapy. Blood.

[16] Xie N, Shen G, Gao W (2023). Neoantigens: promising targets for cancer therapy. Signal Transduct Target Ther.

[17] Lin MJ, Svensson-Arvelund J, Lubitz GS (2022). Cancer vaccines: the next immunotherapy frontier. *Nat Cancer*.

[18] Ott PA, Hu Z, Keskin DB (2017). An immunogenic personal neoantigen vaccine for patients with melanoma. Nature New Biol.

[19] Rojas LA, Sethna Z, Soares KC (2023). Personalized RNA neoantigen vaccines stimulate T cells in pancreatic cancer. Nature New Biol.

[20] Hoenisch Gravel N, Nelde A, Bauer J (2023). TOF_IMS_ mass spectrometry-based immunopeptidomics refines tumor antigen identification. Nat Commun.

[21] Yu F, Teo GC, Kong AT (2023). Analysis of DIA proteomics data using MSFragger-DIA and FragPipe computational platform. Nat Commun.

[22] Declercq A, Bouwmeester R, Hirschler A (2022). MS^2^Rescore: Data-Driven Rescoring Dramatically Boosts Immunopeptide Identification Rates. Mol Cell Proteomics.

[23] Bichmann L, Nelde A, Ghosh M (2019). MHCquant: Automated and Reproducible Data Analysis for Immunopeptidomics. J Proteome Res.

[24] Schuster H, Peper JK, Bösmüller H-C (2017). The immunopeptidomic landscape of ovarian carcinomas. Proc Natl Acad Sci USA.

[25] Bilich T, Nelde A, Bauer J (2020). Mass spectrometry-based identification of a B-cell maturation antigen-derived T-cell epitope for antigen-specific immunotherapy of multiple myeloma. Blood Cancer J.

[26] Marcu A, Bichmann L, Kuchenbecker L (2021). HLA Ligand Atlas: a benign reference of HLA-presented peptides to improve T-cell-based cancer immunotherapy. J Immunother Cancer.

[27] Caron E, Aebersold R, Banaei-Esfahani A (2017). A Case for a Human Immuno-Peptidome Project Consortium. Immunity.

[28] Vizcaíno JA, Kubiniok P, Kovalchik KA (2020). The Human Immunopeptidome Project: A Roadmap to Predict and Treat Immune Diseases. *Molecular & Cellular Proteomics*.

[29] Perez-Riverol Y, Bai J, Bandla C (2022). The PRIDE database resources in 2022: a hub for mass spectrometry-based proteomics evidences. Nucleic Acids Res.

[30] Yi X, Liao Y, Wen B (2021). caAtlas: An immunopeptidome atlas of human cancer. iScience.

[31] Huang X, Gan Z, Cui H (2024). The SysteMHC Atlas v2.0, an updated resource for mass spectrometry-based immunopeptidomics. Nucleic Acids Res.

[32] Scheid J, Lemke S, Hoenisch-Gravel N (2024). MHCquant2 refines immunopeptidomics tumor antigen discovery. In Review.

[33] Mohr C, Gabernet G, Peltzer A (2023). nf-core/epitopeprediction: v2.2.1 - WaldhaeuserOst Hotfix - 2023-03-16. Zenodo.

[34] Vita R, Mahajan S, Overton JA (2019). The Immune Epitope Database (IEDB): 2018 update. Nucleic Acids Res.

[35] Lu Y-C, Parker LL, Lu T (2017). Treatment of Patients With Metastatic Cancer Using a Major Histocompatibility Complex Class II-Restricted T-Cell Receptor Targeting the Cancer Germline Antigen MAGE-A3. J Clin Oncol.

[36] Kunert A, van Brakel M, van Steenbergen-Langeveld S (2016). MAGE-C2-Specific TCRs Combined with Epigenetic Drug-Enhanced Antigenicity Yield Robust and Tumor-Selective T Cell Responses. *J Immunol*.

[37] Loree JM, Wang Y, Syed MA (2021). Clinical and functional characterization of atypical KRAS/NRAS mutations in metastatic colorectal cancer. Clin Cancer Res.

[38] Haridas D, Ponnusamy MP, Chugh S (2014). MUC16: molecular analysis and its functional implications in benign and malignant conditions. FASEB J.

[39] Schuhmacher J, Kleemann L, Richardson JR (2022). Simultaneous Identification of Functional Antigen-Specific CD8+ and CD4+ Cells after In Vitro Expansion Using Elongated Peptides. Cells.

[40] Rammensee H-G, Wiesmüller K-H, Chandran PA (2019). A new synthetic toll-like receptor 1/2 ligand is an efficient adjuvant for peptide vaccination in a human volunteer. J Immunother Cancer.

[41] Aucouturier J, Dupuis L, Deville S (2002). Montanide ISA 720 and 51: a new generation of water in oil emulsions as adjuvants for human vaccines. Expert Rev Vaccines.

[42] Rammensee H-G, Gouttefangeas C, Heidu S (2021). Designing a SARS-CoV-2 T-Cell-Inducing Vaccine for High-Risk Patient Groups. Vaccines (Basel).

[43] Turtle CJ, Hay KA, Hanafi L-A (2017). Durable Molecular Remissions in Chronic Lymphocytic Leukemia Treated With CD19-Specific Chimeric Antigen Receptor-Modified T Cells After Failure of Ibrutinib. J Clin Oncol.

[44] Zhao Q, Ahmed M, Tassev DV (2015). Affinity maturation of T-cell receptor-like antibodies for Wilms tumor 1 peptide greatly enhances therapeutic potential. Leukemia.

[45] Becker JP, Riemer AB (2022). The Importance of Being Presented: Target Validation by Immunopeptidomics for Epitope-Specific Immunotherapies. Front Immunol.

[46] Kote S, Pirog A, Bedran G (2020). Mass Spectrometry-Based Identification of MHC-Associated Peptides. Cancers (Basel).

[47] Weinzierl AO, Lemmel C, Schoor O (2007). Distorted Relation between mRNA Copy Number and Corresponding Major Histocompatibility Complex Ligand Density on the Cell Surface. *Molecular & Cellular Proteomics*.

[48] Fortier M-H, Caron E, Hardy M-P (2008). The MHC class I peptide repertoire is molded by the transcriptome. J Exp Med.

[49] Walz S, Stickel JS, Kowalewski DJ (2015). The antigenic landscape of multiple myeloma: mass spectrometry (re)defines targets for T-cell–based immunotherapy. Blood.

[50] Vansteenkiste J, Zielinski M, Linder A (2013). Adjuvant MAGE-A3 immunotherapy in resected non-small-cell lung cancer: phase II randomized study results. J Clin Oncol.

[51] Clifton GT, Hale D, Vreeland TJ (2020). Results of a Randomized Phase IIb Trial of Nelipepimut-S + Trastuzumab versus Trastuzumab to Prevent Recurrences in Patients with High-Risk HER2 Low-Expressing Breast Cancer. Clin Cancer Res.

[52] Kacen A, Javitt A, Kramer MP (2022). Uncovering the modified immunopeptidome reveals insights into principles of ptm-driven antigenicity. Cancer Biology.

[53] Backert L, Kowalewski DJ, Walz S (2017). A meta-analysis of HLA peptidome composition in different hematological entities: entity-specific dividing lines and “pan-leukemia” antigens. Oncotarget.

[54] Wacker M, Bauer J, Wessling L (2023). Immunoprecipitation methods impact the peptide repertoire in immunopeptidomics. Front Immunol.

[55] Tu C, Li J, Shen S (2016). Performance Investigation of Proteomic Identification by HCD/CID Fragmentations in Combination with High/Low-Resolution Detectors on a Tribrid, High-Field Orbitrap Instrument. PLoS ONE.

[56] Parker R, Tailor A, Peng X (2021). The Choice of Search Engine Affects Sequencing Depth and HLA Class I Allele-Specific Peptide Repertoires. Mol Cell Proteomics.

[57] Segerman B (2020). The Most Frequently Used Sequencing Technologies and Assembly Methods in Different Time Segments of the Bacterial Surveillance and RefSeq Genome Databases. Front Cell Infect Microbiol.

[58] Löffler MW, Mohr C, Bichmann L (2019). Multi-omics discovery of exome-derived neoantigens in hepatocellular carcinoma. Genome Med.

[59] Weinstein JN, Collisson EA, Mills GB (2013). The Cancer Genome Atlas Pan-Cancer analysis project. Nat Genet.

[60] Wilhelm M, Zolg DP, Graber M (2021). Deep learning boosts sensitivity of mass spectrometry-based immunopeptidomics. Nat Commun.

[61] Heitmann JS, Bilich T, Tandler C (2022). A COVID-19 peptide vaccine for the induction of SARS-CoV-2 T cell immunity. Nature New Biol.

[62] Andreou M, Kyprianidou M, Cortas C (2023). Prognostic Factors Influencing Survival in Ovarian Cancer Patients: A 10-Year Retrospective Study. Cancers (Basel).

[63] Halabi S, Kelly WK, Ma H (2016). Meta-Analysis Evaluating the Impact of Site of Metastasis on Overall Survival in Men With Castration-Resistant Prostate Cancer. J Clin Oncol.

[64] Sahin U, Derhovanessian E, Miller M (2017). Personalized RNA mutanome vaccines mobilize poly-specific therapeutic immunity against cancer. Nature New Biol.

[65] Almeida LG, Sakabe NJ, deOliveira AR (2009). CTdatabase: a knowledge-base of high-throughput and curated data on cancer-testis antigens. Nucleic Acids Res.

[66] Mohr C, Friedrich A, Wojnar D (2018). qPortal: A platform for data-driven biomedical research. PLoS One.

[67] Ewels PA, Peltzer A, Fillinger S (2020). The nf-core framework for community-curated bioinformatics pipelines. Nat Biotechnol.

[68] Nelde A, Kowalewski DJ, Stevanović S (2019). Purification and identification of naturally presented MHC class I and II ligands. Antigen Processing: Methods and Protocols.

[69] Toprak UH, Gillet LC, Maiolica A (2014). Conserved Peptide Fragmentation as a Benchmarking Tool for Mass Spectrometers and a Discriminating Feature for Targeted Proteomics. *Molecular & Cellular Proteomics*.

[70] Schöllhorn A, Schuhmacher J, Besedovsky L (2021). Integrin Activation Enables Sensitive Detection of Functional CD4^+^ and CD8^+^ T Cells: Application to Characterize SARS-CoV-2 Immunity. Front Immunol.

[71] Walz JS (2025). Created in BioRender.

[72] Walz JS (2025). Created in BioRender.

[73] Lemke S, Dubbelaar ML, Zimmermann P (2025). PCI-db: a novel primary tissue immunopeptidome database to guide next-generation peptide-based immunotherapy development. In Review.

