# Supplementary material for: PCI-DB: a novel primary tissue immunopeptidome database to guide next-generation peptide-based immunotherapy development
Source: J Immunother Cancer. 2025 Apr 15;13(4):e011366. doi: 10.1136/jitc-2024-011366 (PMC12001369; doi:10.1136/jitc-2024-011366)
Supplement: online supplemental figure 1 [file jitc-13-4-s003.pdf]

Supplementary Material

for

PCI-DB: A novel primary tissue immunopeptidome database to guide next-generation peptide-based immunotherapy development

Steffen Lemke, Marissa L. Dubbelaar, Patrick Zimmermann, Jens Bauer, Annika Nelde, Naomi Hoenisch-Gravel, Jonas Scheid, Marcel Wacker, Susanne Jung, Anna Dengler, Yacine Maringer, Hans-Georg Rammensee, Cécile Gouttefangeas, Sven Fillinger, Tatjana Bilich, Jonas S. Heitmann, Sven Nahnsen, Juliane S. Walz*

*Correspondence to: juliane.walz@med.uni-tuebingen.de

a

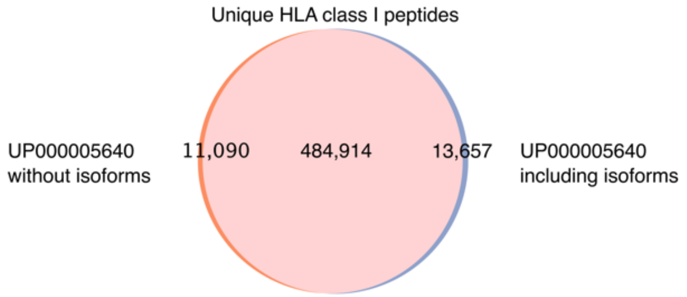

b

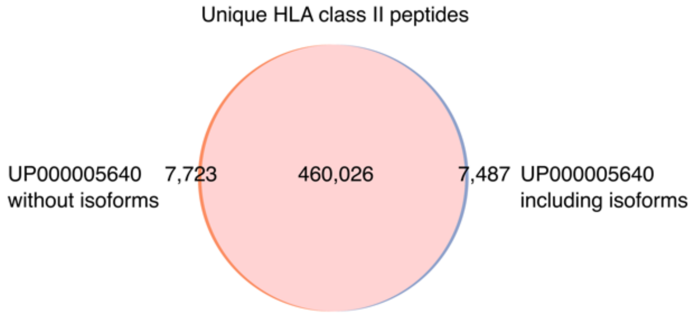

Fig. S1. Overlap analysis of unique HLA peptides upon including isoforms to the reference proteome. a,b, Comparison of unique HLA class I **(a)** and class II **(b)** peptide sequences detected using the UP000005640 reference proteome, with and without isoforms. Abbreviations: HLA, human leukocyte antigen

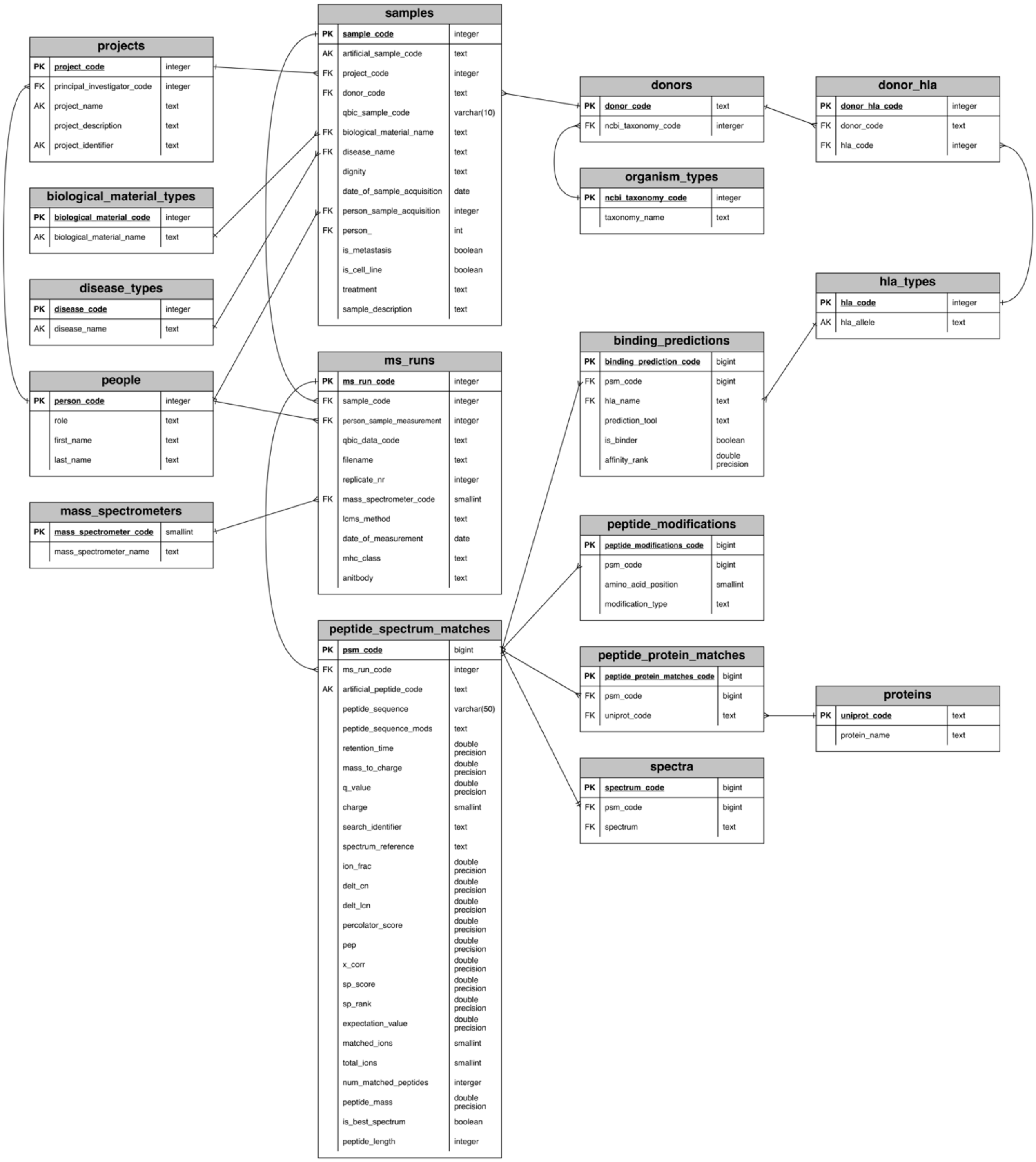

Fig. S2. Entity relationship diagram of the PCI-DB. Table names are highlighted in gray, with columns and corresponding data types listed below. Information on primary, foreign, and alternate keys is depicted in each database table's first column. Abbreviations: PK, primary key; FK, foreign

key; AK, alternate key; MS, mass spectrometry; HLA, human leukocyte antigen; psm, peptide-spectrum match

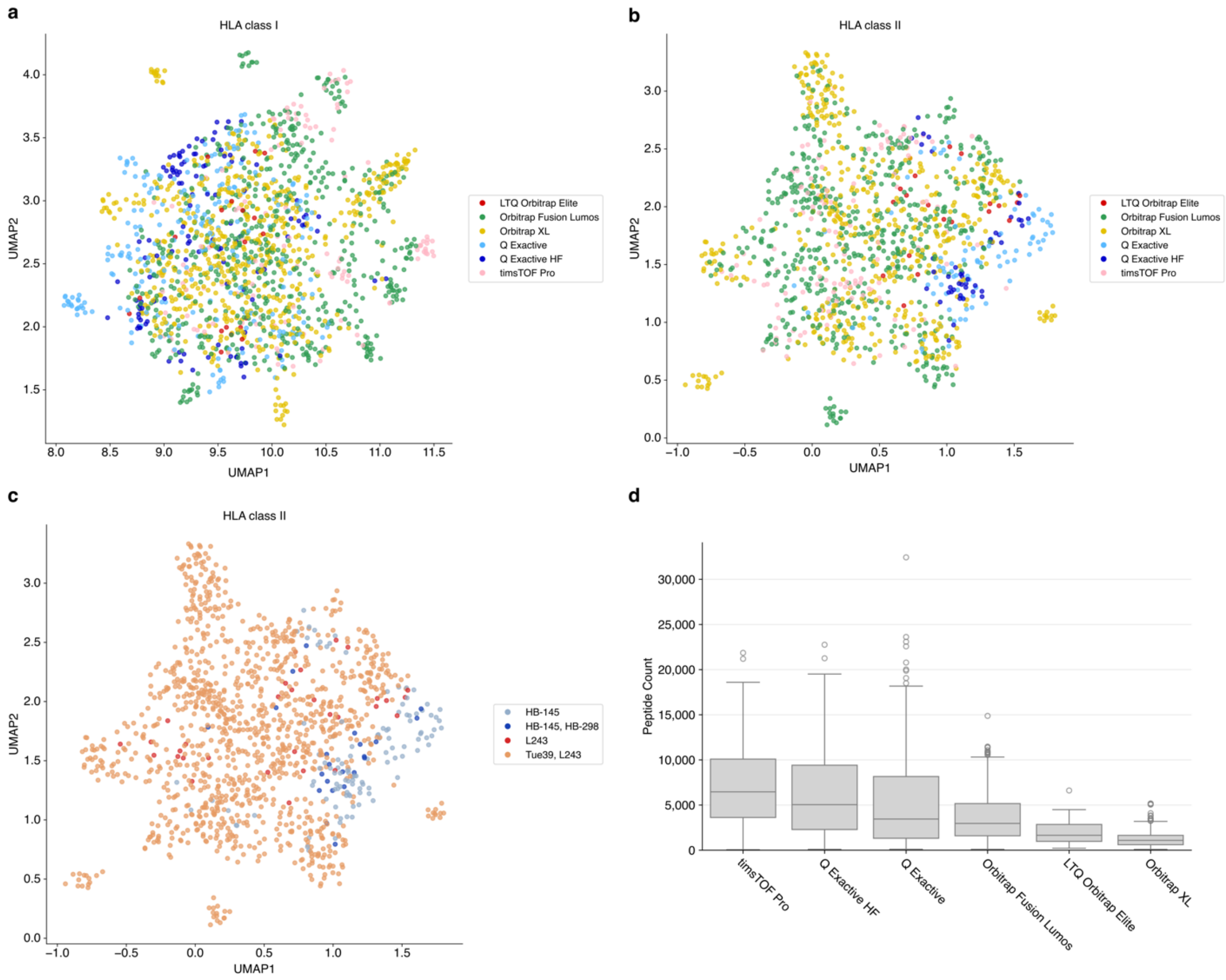

Fig. S3. Analysis of mass spectrometer and sample preparation bias. a,b,c, UMAP projection of HLA class I **(a)** and HLA class II **(b,c)** samples based on the overlap coefficient computed pairwise between the samples. Mass spectrometers **(a,b)** and used antibodies in sample preparation **(c)** are indicated by color. **d,** The number of identified peptides per sample is shown across different mass spectrometers. Abbreviations: UMAP, Uniform Manifold Approximation and Projection; HLA, human leukocyte antigen

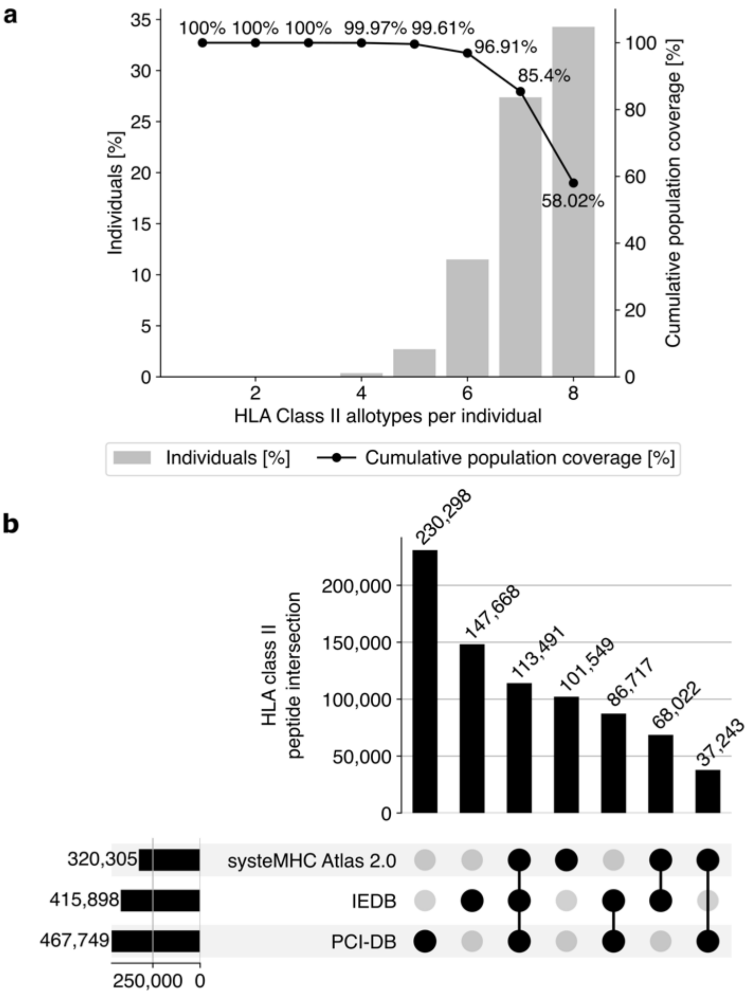

Fig. S4. PCI-DB HLA class II peptide content benchmark and allele population coverage. a, Global population coverage of the HLA class II alleles contained in the database. The grey bars display the percentage of the population that has the number of alleles in the database (left y-axis). The black dots show the cumulative population coverage in percent (right y-axis). **b,** Overlap analysis of the unique HLA class II peptides shared between PCI-DB, IEDB, and the available data of the systeMHC Atlas 2.0. Abbreviations: HLA, Human leukocyte antigen; PCI-DB, peptides for cancer immunotherapy database; IEDB, immune Epitope Database

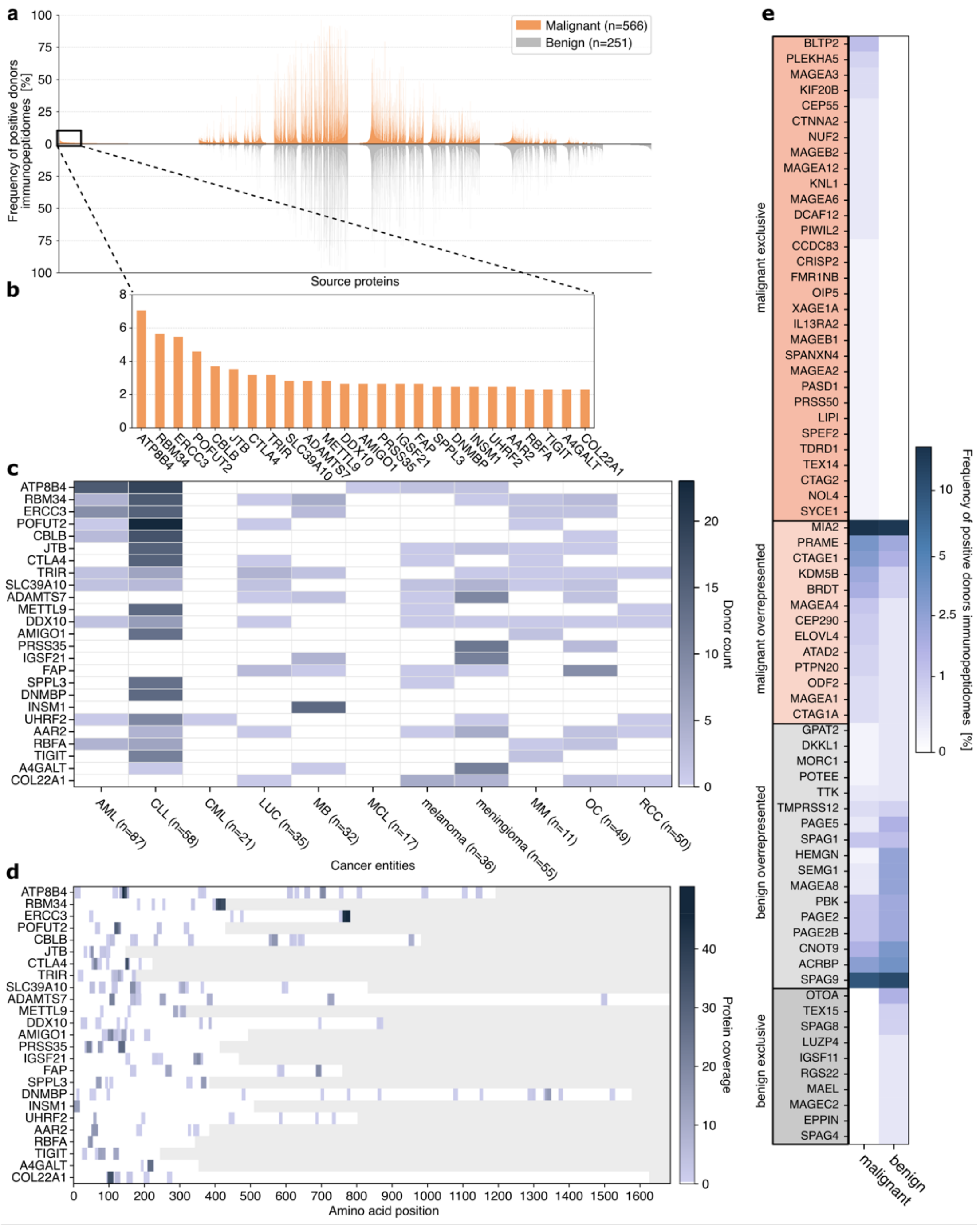

Fig. S5. Characterization of tumor-associated proteins in the HLA class II immunopeptidomes of the PCI-DB. a, Comparative profiling of HLA class II source proteins of malignant and benign tissue samples. The frequency of positive donor immunopeptidomes is shown on the y-axis, and the source proteins on the x-axis. Proteins with a representation frequency

in the immunopeptidome of less than 0.5% in the malignant and benign cohort were omitted from this analysis. **b,** The 25 most frequently found malignant exclusive proteins in HLA class II in all donors. **c,** Frequency of HLA class II peptides derived from the 25 most abundant malignant-exclusive proteins in cancer patients (n) in different cancer samples. **d,** Protein coverage of the malignant exclusive proteins. The coverage of each amino acid position describes the number of times that amino acid was detected as part of a peptide in an immunopeptidome sample. **e,** Analysis of CTAs represented within the HLA class II immunopeptidome. Percentage of donors with positive identification of HLA class II peptides originating from CTAs are shown. CTAs were grouped in four categories: malignant exclusive, benign exclusive (if only peptides from the respective CTAs could be found in samples from malignant/benign donors), and malignant/benign overrepresented (more peptides from malignant/benign donors were found than in the other group). Abbreviations: HLA, human leukocyte antigen; CTA cancer-testis antigen; AML, acute myeloid leukemia; CLL, chronic lymphocytic leukemia; CML, chronic myeloid leukemia; LUC, lung cancer; MB, medulloblastoma; MCL, mantle cell lymphoma; MM, multiple myeloma; OC, ovarian carcinoma; OPSCC, oropharyngeal squamous cell carcinoma; RCC, renal cell carcinoma

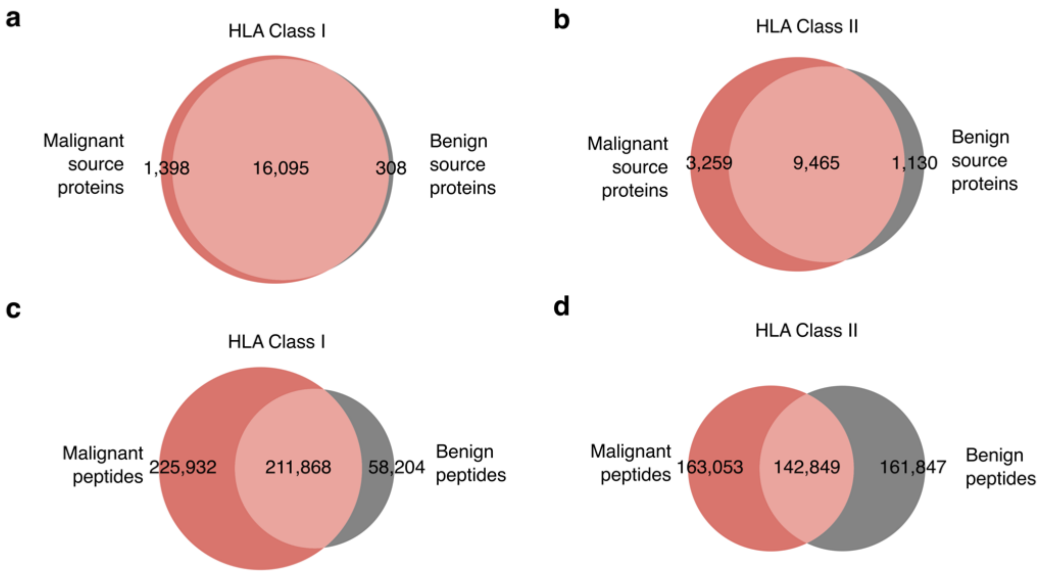

Fig. S6. Overlap analysis of HLA class I/II peptides and their source proteins. a,b, Overlap analysis of source proteins as origin of detected HLA class I **(a)** and HLA class II **(b)** peptides in malignant and benign tissue. **c,d,** Overlap of HLA class I **(c)** and class II **(d)** peptides in malignant and benign tissue. Abbreviations: HLA, human leukocyte antigen

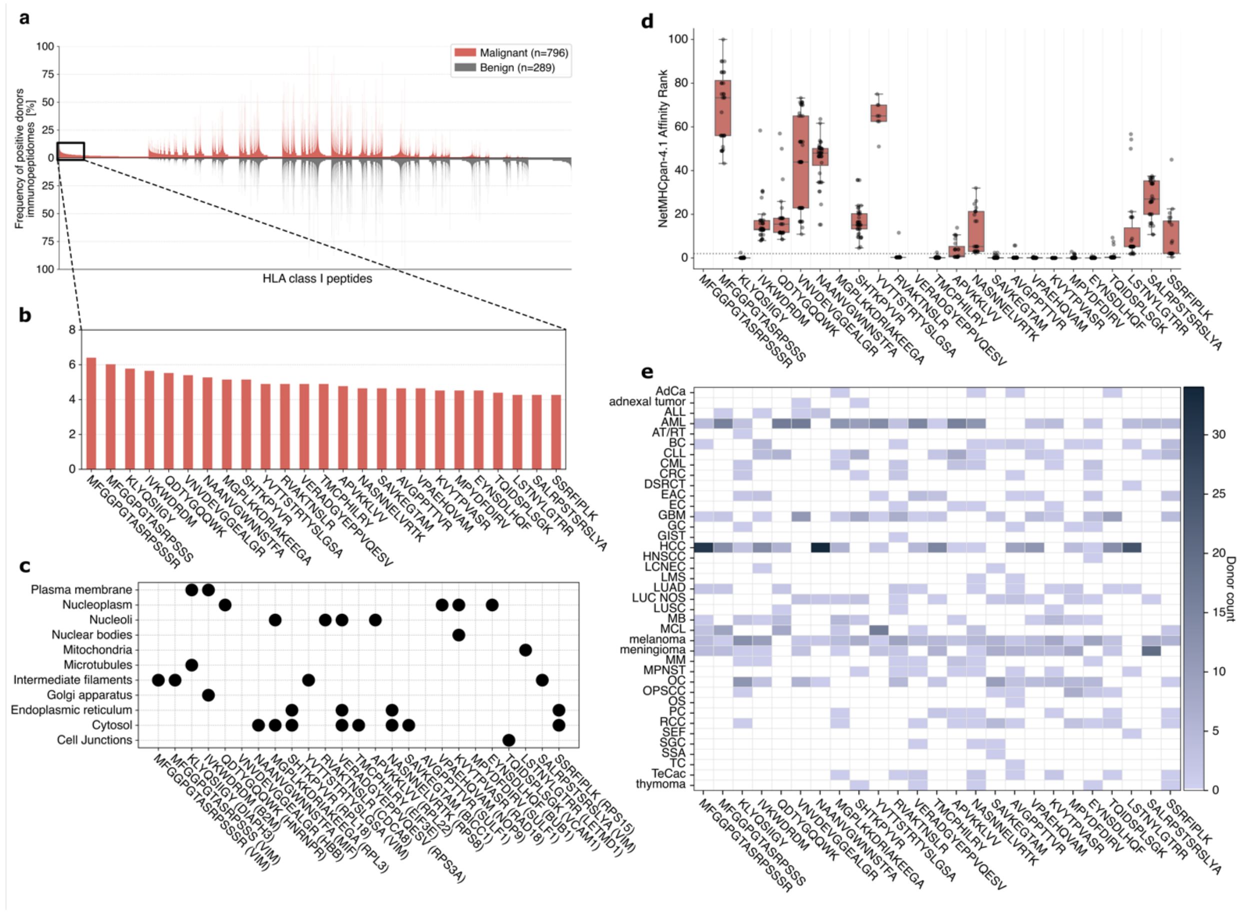

Fig. S7. Characterization of malignant-exclusive HLA class I peptides within the PCI-DB. a, Comparative profiling of HLA class I peptides identified in malignant and benign tissue samples. The frequency of positive donor immunopeptidomes is shown on the y-axis, and the peptides on the x-axis. Peptides with a frequency of less than 0.5% in the malignant and benign cohort were omitted from this analysis. **b,** Magnification of the 25 most frequently detected malignant exclusive HLA class I peptides across all cancer entities. **c,** Subcellular location of the source proteins of the most frequently detected malignant exclusive peptides in HLA class I. **d,** HLA binding prediction using NetMHCpan-4.1. For each peptide identification, the corresponding donor's HLA allotypes were used for binding prediction. The dashed line at the NetMHCpan-4.1 affinity rank of 2 represents the threshold for binders. Missing values for HLA class I peptide

binding predictions are due to NetMHCpan-4.1 not supporting affinity predictions for peptides of length >14. **e,** Number of positive donors in different cancer entities for each HLA class I peptide. Abbreviations: HLA, Human leukocyte antigen; AdCa, adrenal cancer; ALL, acute lymphocytic leukemia; AML, acute myeloid leukemia; AT/RT, atypical teratoid rhabdoid tumor; BC, breast cancer; CLL, chronic lymphocytic leukemia; CML, chronic myeloid leukemia; CRC, colorectal cancer; EAC, esophageal carcinoma; EC, endometrial cancer; GBM, glioblastoma; GC, gastric carcinoma; GIST, gastrointestinal stromal tumor; HCC, hepatocellular carcinoma; HNSCC, head and neck squamous cell carcinoma; LCNEC, large cell neuroendocrine carcinoma; LMS, leiomyosarcoma; LUAD, lung adenocarcinoma; LUC NOS, lung cancer; LUSC, lung squamous cell carcinoma; MB, medulloblastoma; MCL, mantle cell lymphoma; MM, multiple myeloma; MPNST, malignant peripheral nerve sheath tumor, OC, ovarian carcinoma; OPSCC, oropharyngeal squamous cell carcinoma; OS, osteosarcoma; PC, pancreatic carcinoma; RCC, renal cell carcinoma; SGC, salivary gland cancer; SSA, synovial sarcoma; TC, thyroid cancer; TeCac, testicular cancer

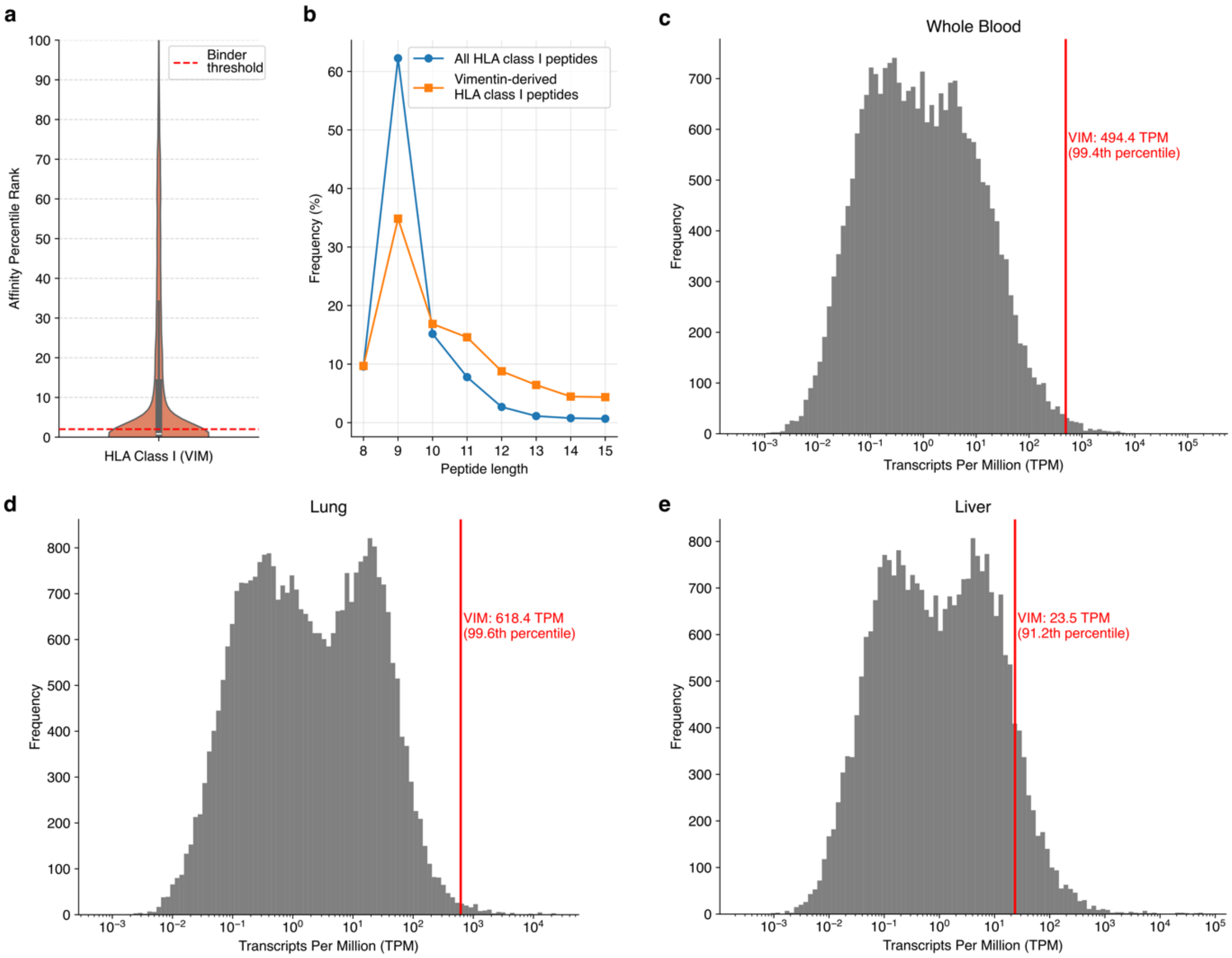

Fig. S8. Evaluation of HLA peptides originating from Vimentin. a, Binding prediction scores of Vimentin-derived peptides in HLA class I samples computed by NetMHCpan-4.1. **b,** Length distribution of all HLA class I peptides and Vimentin-derived HLA class I peptides. **c,d,e,** Gene expression from GTEx in transcripts per million (TPM) of all genes shown for Whole Blood **(a)**, Lung **(b)**, and Liver **(c)**. The red line indicates the TPM values for Vimentin (VIM).

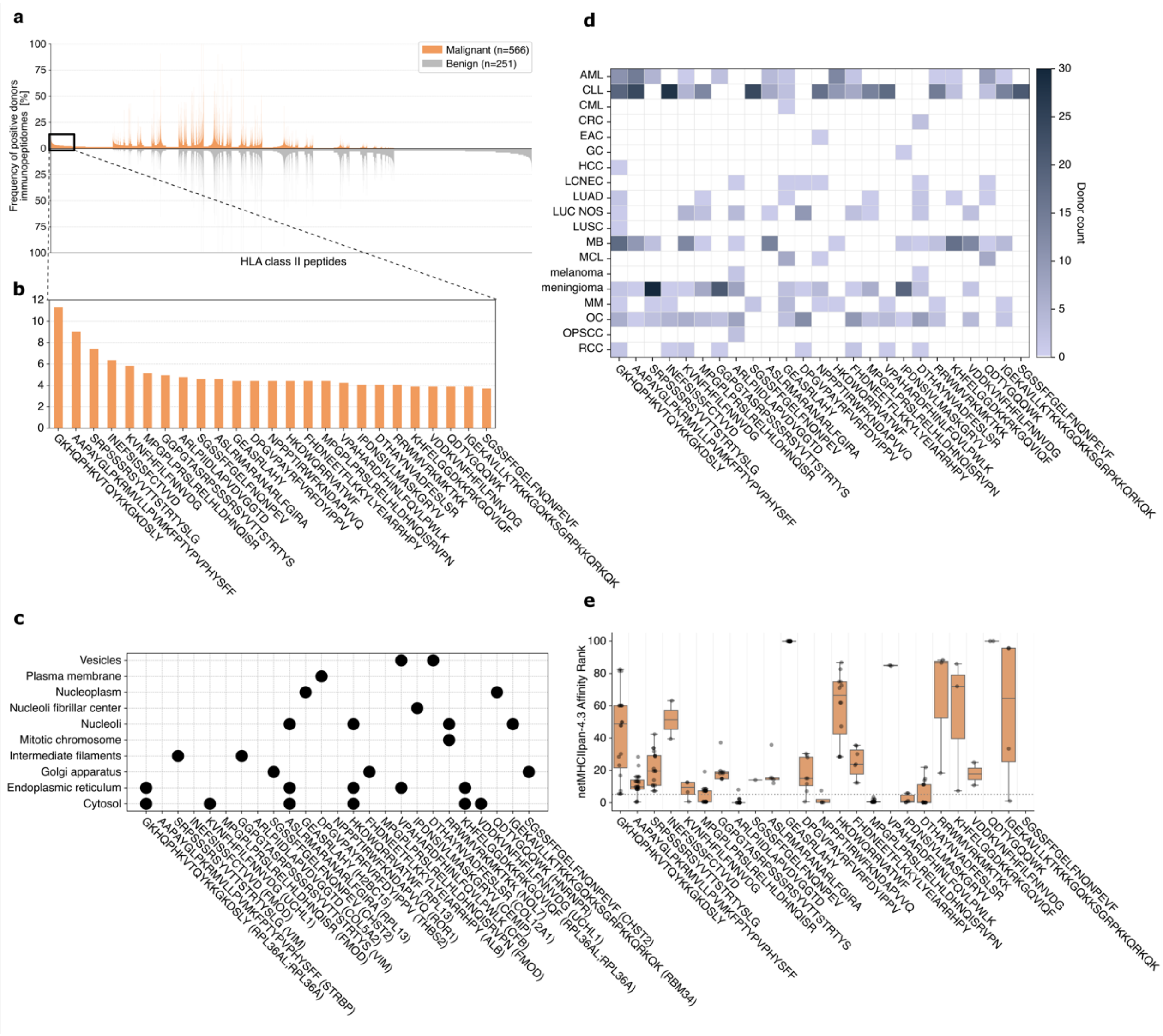

Fig. S9. Characterization of malignant-exclusive HLA class II peptides within the PCI-DB. **a,** Comparative profiling of HLA class II peptides identified in malignant and benign tissue samples. The frequency of positive donor immunopeptidomes is shown on the y-axis, and the peptides on the x-axis. Peptides with a frequency of less than 0.5% in the malignant and benign cohort were omitted from this analysis. **b,** Magnification of the 25 most frequently detected malignant exclusive HLA class II peptides across all cancer entities. **c,** Subcellular location of the source proteins of the most frequently detected malignant exclusive peptides in HLA class II. **d,**

Number of positive donors in different cancer entities for each HLA class II peptide. **e,** HLA binding prediction using NetMHCIIpan-4.3. For each peptide identification, the corresponding donor's HLA allotypes were used for binding prediction. The dashed line at the NetMHCIIpan-4.3 affinity rank of 2 represents the threshold for binders. Abbreviations: HLA, Human leukocyte antigen; AML, acute myeloid leukemia; CLL, chronic lymphocytic leukemia; CRC, colorectal cancer; EAC, esophageal carcinoma; GC, gastric carcinoma; HCC, hepatocellular carcinoma; LCNEC, large cell neuroendocrine carcinoma; LUAD, lung adenocarcinoma; LUC NOS, lung cancer; LUSC, lung squamous cell carcinoma; MB, medulloblastoma; MCL, mantle cell lymphoma; MM, multiple myeloma; OC, ovarian carcinoma; OPSCC, oropharyngeal squamous cell carcinoma; RCC, renal cell carcinoma

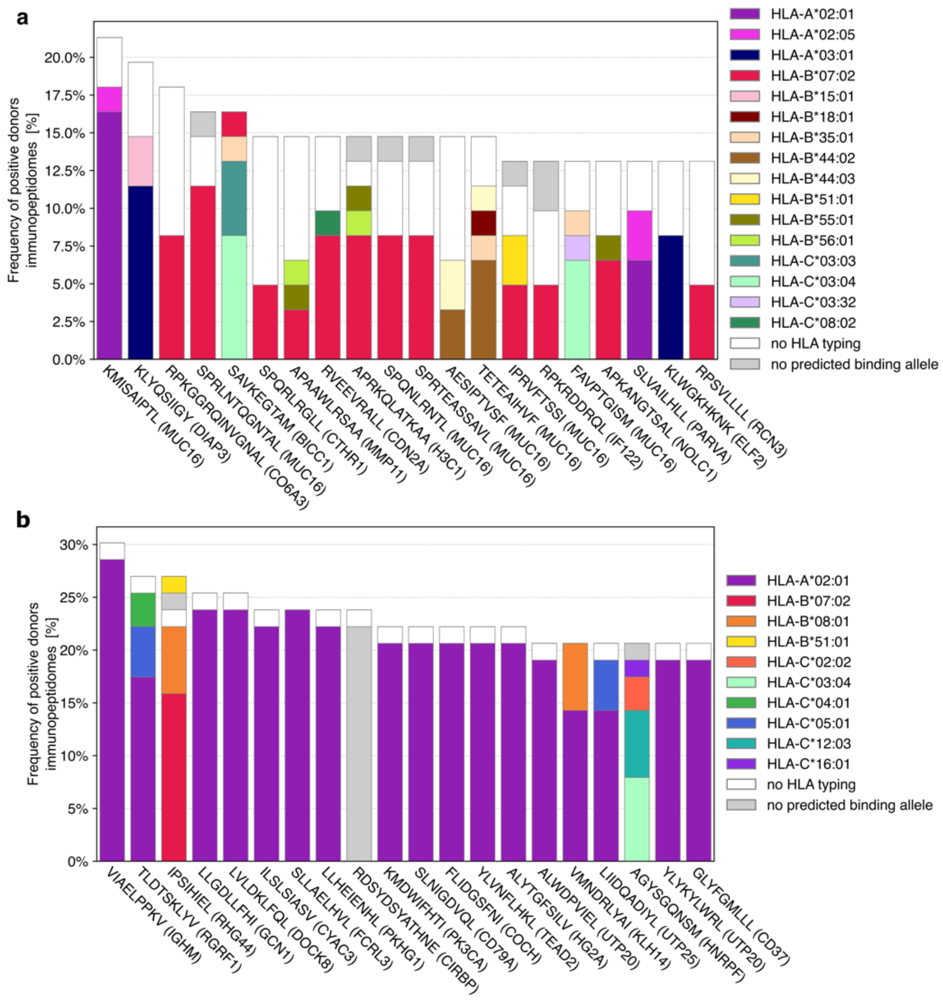

Fig. S10. HLA allotype assignment of malignant-exclusive HLA class I in OvCa and CLL cohort. a,b, Stacked bar plots depicting the most abundant malignant-exclusive peptides in the OvCa (**a**) and CLL (**b**) cohort, annotated with their corresponding source proteins. Each colored sub-bar indicates the frequency of positive donors with positive binder prediction by NetMHCpan-4.1 for the respective HLA-allele. Abbreviations: HLA, human leukocyte antigen; OvCa, ovarian carcinoma; CLL, chronic lymphocytic leukemia

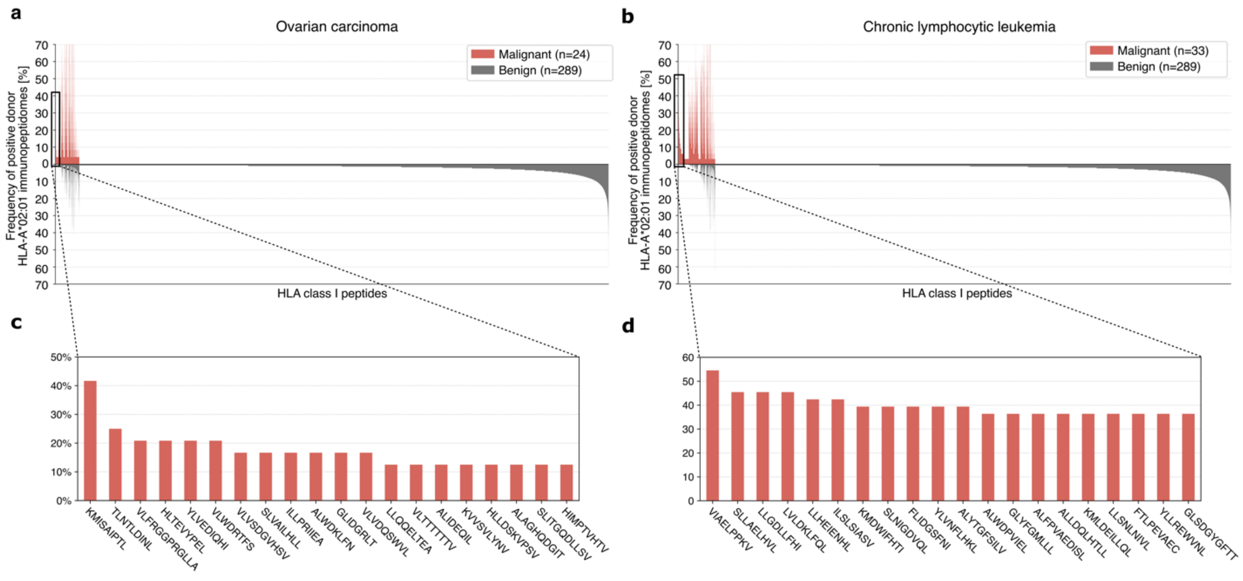

Fig. S11. Peptide target identification of HLA-A*02:01 peptides for cancer immunotherapy. a,b, Comparative profiling of HLA-A*02:01-positive immunopeptidomes restricted to HLA-A*02:01 ligands of malignant OvCa **(a)** and CLL **(b)** samples compared to all benign data in the PCI-DB. Frequency of positive donor immunopeptidomes (y-axis) for each HLA class II peptide (x-axis). Peptides with a frequency of less than 0.5% in the malignant and benign cohort were omitted from this analysis. **c,d,** Magnification of the most abundant ovarian carcinoma-exclusive HLA-A*02:01 peptides **(c)** and chronic lymphocytic leukemia-exclusive peptides **(d)**. Abbreviations: HLA, human leukocyte antigen; OvCa, ovarian carcinoma; CLL, chronic lymphocytic leukemia

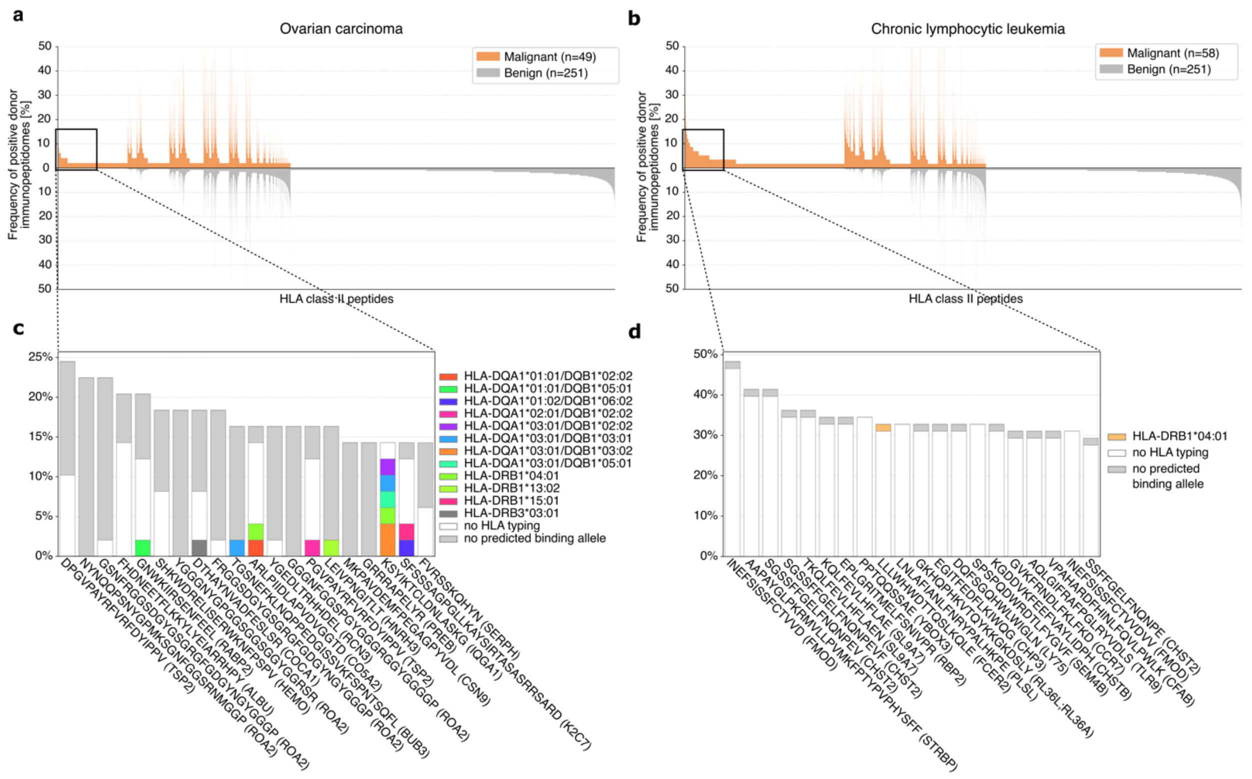

Fig. S12. Peptide target identification of HLA class II peptides for cancer immunotherapy. a,b, Comparative profiling of HLA class II immunopeptidomes of malignant OvCa **(a)** and CLL **(b)** samples compared to all benign data in the PCI-DB. Frequency of positive donor immunopeptidomes (y-axis) for each HLA class II peptide (x-axis). Peptides with a frequency of less than 0.5% in the malignant and benign cohort were omitted from this analysis. **c,d,** Magnification of the most abundant ovarian carcinoma-exclusive peptides **(c)** and chronic lymphocytic leukemia-exclusive peptides **(d)**, annotated with their corresponding source proteins. Each colored sub-bar indicates the frequency of positive donors with positive binder prediction by NetMHCIIpan-4.3 for the respective HLA-alleles. Abbreviations: HLA, human leukocyte antigen; OvCa, ovarian carcinoma; CLL, chronic lymphocytic leukemia

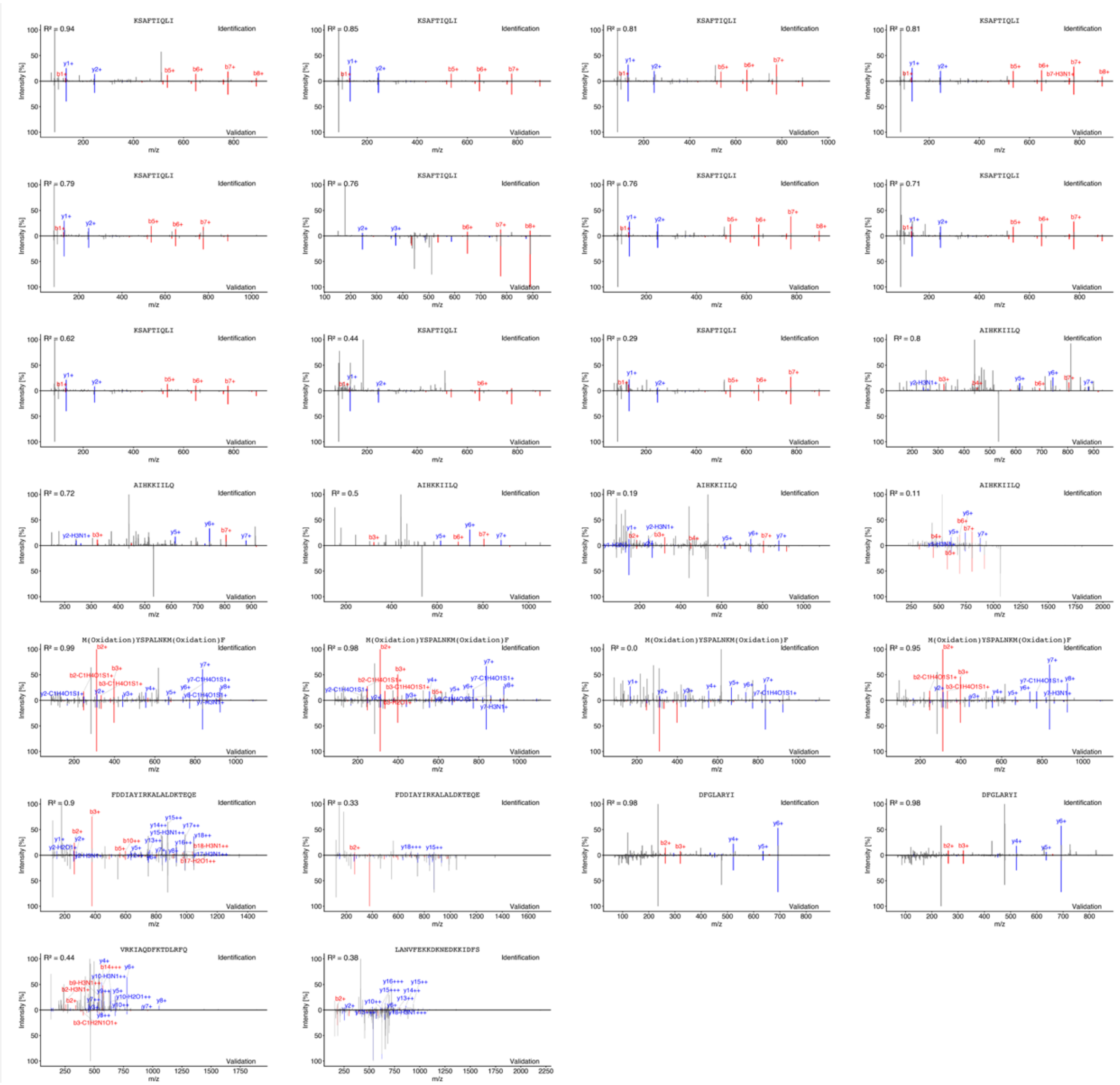

Fig. S13. Mirror plots for synthetic peptide-based validation of neoepitope candidates. Fragment spectra of experimentally eluted peptides are shown above the x-axes (Identification), and the corresponding synthetic peptide spectra are shown below the x-axes (Validation). Matching b- and y-ions are displayed in red and blue, respectively. Each mirror plot displays the synthetic peptide comparison's calculated spectral correlation coefficient (R^2).

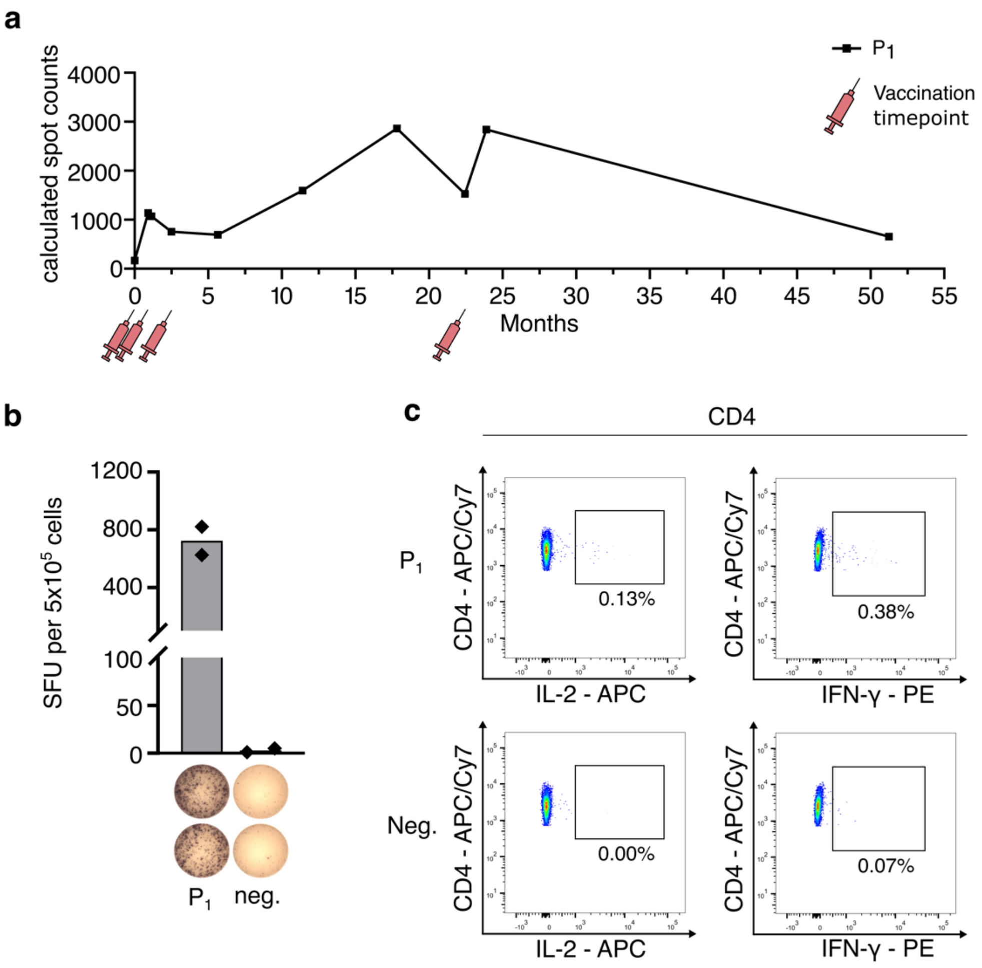

Fig. S14. Personalized therapeutic vaccination using a neoepitope identified in the PCI-DB. A patient with metastatic prostate cancer (ProCa02) was vaccinated with the neoepitope FDDIAYIRK**A**LALDKTEQE (PIK3CA T1025A). This neoepitope was identified as naturally presented peptide target in the global neoepitope search within the PCI-DB. The specific PK3CA T1025A mutation was confirmed by gene panel sequencing of the patient's tumor. Four vaccine doses were administered subcutaneously. Vaccine peptide was adjuvanted with the TLR1/2-ligand XS15 and emulsified in Montanide ISA 51 VG. **a,** Longitudinal analysis of neoepitope-specific T-cell response assessed by IFN-γ ELISpot assay after 12 day *in vitro* expansion in technical duplicates. Calculated spot counts are depicted on the y-axis. Vaccination time points are indicated

by red syringes. **b,** Exemplary IFN-γ ELISpot assay results six weeks after the fourth vaccination for the neoepitope peptide and negative control in technical duplicates. **c,** Flow cytometry-based functional characterization of peptide-specific CD4+ T cells 28 months after the fourth vaccination, following 12-day *in-vitro* stimulation and intracellular cytokine staining of IFN-γ and IL-2. Abbreviations: PCI-DB, peptides for cancer immunotherapy database; IFN-γ, Interferon-γ; ELISpot, Enzyme-linked immuno spot; IL-2, Interleukin-2; CD4, cluster of differentiation 4; SFU, spot forming units

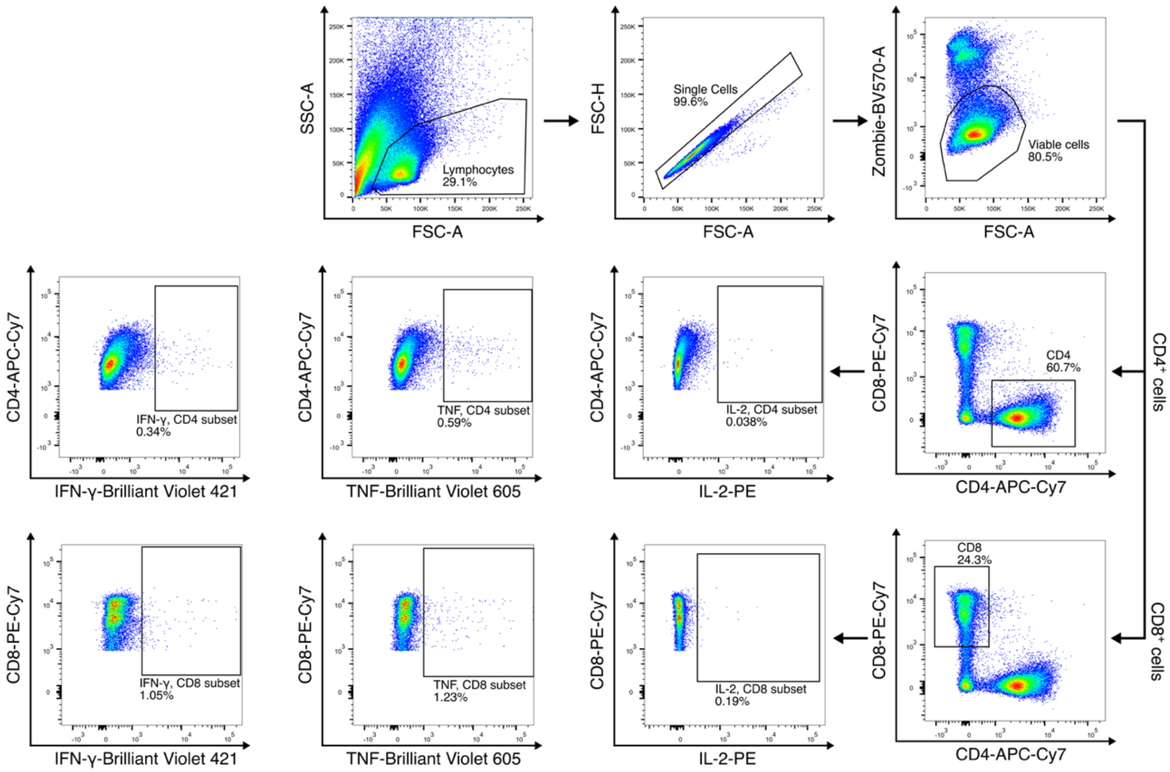

Fig. S15. Gating strategy for ICS evaluation of peptide-specific CD4$^+$ and CD8$^+$ T cells All measured cells were first gated for the lymphocyte population (FSC-A vs. SSC-A), followed by gating for single cells (FSC-A vs. FSC-H) and then viable cells (FSC-A vs. Zombie-BV570-A). In the next step, the viable cell population was gated for CD4$^+$ and CD8$^+$ cell populations (CD4-APC-Cy7 vs. CD8-PE-Cy7). Those two cell populations were then independently gated for IFN-γ (IFN-γ-Brilliant Violet 421), TNF (TNF-Brilliant Violet 605), and IL-2 (IL-2-PE).

Peptide ID	P1	P2	P3
**Detected Peptide Sequence**	SPQNLRNTL	ALHSHMINK	ALASGTGLFK
**Source Protein**	Mucin-16	Periostin	Prolyl endopeptidase FAP
**HLA Restriction**	B*07:02	A*03:01	A*03:01
**Peptide length**	9	9	10
**Malignant seq. in PCI-DB**	10	52	22
**Malignant seq. in OvCa in PCI-DB**	10	6	4
**Benign origin seq. in PCI-DB**	0	0	0
**Vaccinated sequence**	MDTNLEPVTR**SPQNLRNTL**	ESNVNVELLN**ALHSHMINK**	SYGGYVSSL**ALASGTGLFK**

Table S1. Overview of vaccinated HLA class I peptides in the OvCa01 patient.
