# Supplementary material for: PCI-DB: a novel primary tissue immunopeptidome database to guide next-generation peptide-based immunotherapy development
Source: J Immunother Cancer. 2025 Apr 15;13(4):e011366. doi: 10.1136/jitc-2024-011366 (PMC12001369; doi:10.1136/jitc-2024-011366)
Supplement: online supplemental file 3 [file jitc-13-4-s004.pdf]

Supplementary Material

for

PCI-DB: A novel primary tissue immunopeptidome database to guide next-generation peptide-based immunotherapy development

Steffen Lemke, Marissa L. Dubbelaar, Patrick Zimmermann, Jens Bauer, Annika Nelde, Naomi Hoenisch-Gravel, Jonas Scheid, Marcel Wacker, Susanne Jung, Anna Dengler, Yacine Maringer, Hans-Georg Rammensee, Cécile Gouttefangeas, Sven Fillinger, Tatjana Bilich, Jonas S. Heitmann, Sven Nahnsen, Juliane S. Walz*

*Correspondence to: juliane.walz@med.uni-tuebingen.de

Supplementary Methods

Database search

MS data were processed using the nf-core/mhcquant pipeline (release 2.6.0)[1-3]. HLA class I peptides were searched using a digest mass range of 800-2,500 m/z with a precursor charge of 2-3 and a peptides length filter of 8-15 amino acids, while HLA class II peptides were searched using a digest mass range of 800-5,000 m/z with a precursor charge of 2-5 and a length filter of 8-30 amino acids. Methionine oxidation and cystine carbaminomethylation were specified as variable modifications. Reductive alkylation is not always performed in immunopeptidomics experiments; however, various projects included in this study added iodoacetamide during sample preparation. Therefore, cysteine carbamidomethylation was included as a variable modification in the peptide search to allow for homogenous data processing. Different settings for MHCquant2 were used depending on the mass spectrometer, the mass analyzer, and the fragmentation method. A detailed list of all mass spectrometer-specific parameters can be found in Data S2. All other parameters were kept at default values. MHCquant2 uses Comet (v. 2023.01 rev. 0) as a search engine [4]. The reviewed human reference proteome UP000005640 (release 2024_01) with and without protein isoforms was used as protein database. Rescoring was performed using Percolator [5] at a 1% peptide FDR for each sample. Rescoring was boosted using MS^{2}Rescore, implementing MS^{2}PIP for peak intensity prediction and DeepLC for retention time prediction[6-8]. A global FDR of 1% on peptide level was computed for both the HLA class I and HLA class II sample subsets using Percolator to limit the accumulation of false positive peptide identifications. This process resulted in peptide lists for HLA class I and HLA class II under the specified global FDR, which was then utilized to filter the peptide identifications in each sample. After global FDR filtering, 60 samples with the

corresponding detected peptides were removed from the dataset to avoid overlaps with future publications. These data will then be submitted in a database update.

Neoepitope search

The 1000 most frequent missense mutations across all TCGA [9] cohorts were obtained through the Genomic Data Commons (GDC) portal (https://portal.gdc.cancer.gov/, accessed 07.05.24), and these altered amino acid sequences were added to the reference protein database UP000005640. The number of mutations included in the search was based on a trade-off between covering the most frequent missense variants and limiting the inflation of the search space to prevent random chance identifications. All samples were searched using the neoepitope-containing reference database using the settings described in the previous subsection and filtered using a global FDR of 1%. A neoepitope was considered to be identified when the peptide sequence exclusively matched a sequence containing a missense mutation and did not correspond to any wild-type sequence.

HLA binding prediction

HLA binding prediction was performed using the nf-core/epitopeprediction pipeline [10]. If four-digit HLA-typing information was available for the respective donor, all identified peptides were predicted for HLA binding using NetMHCpan-4.1 for HLA class I peptides or NetMHCIIpan-4.3 for HLA class II peptides [11]. Peptides were considered binders if the affinity rank was equal to or less than 2% for NetMHCpan-4.1 or 5% for NetMHCIIpan-4.3.

Analysis of peptides by LC-MS/MS

Peptide samples measured on Orbitrap devices were measured by reversed-phase liquid chromatography (LC, nanoUHPLC, UltiMate 3000 RSLCnano, Thermo Fisher, Waltham, MA, USA) and subsequent analysis in an online coupled Orbitrap Fusion Lumos mass spectrometer

(Thermo Fisher) or Q Exactive HF mass spectrometer (Thermo Fisher). Peptide separation was performed at 50 °C at a flow rate of 300 nL/min on a 50 μm × 25 cm separation column (PepMap C18, Thermo Fisher), applying a gradient ranging from 2.4% to 32.0% acetonitrile (ACN) over 90 min. Eluting peptides were ionized *via* nanospray ionization and analyzed in the mass spectrometer using data-dependent acquisition (DDA) mode employing a top-speed collisional-induced dissociation (CID, HLA class I peptides, normalized collision energy 35%) or higher-energy collisional dissociation (HCD, HLA class II peptides, normalized collision energy 30%) fragmentation method on Lumos or a top 35 HCD method on Q Exactive HF (HLA class I peptides, normalized collision energy 25%, HLA class II peptides, normalized collision energy 23%), generating fragment spectra with a resolution of 30,000 (Lumos) or 45,000 (Q Exactive HF), a mass range limited to 400-650 m/z for HLA class I peptides and 400-1,000 m/z for HLA class II peptides, positive charge states 2–3 for HLA class I and 2–5 for HLA class II were selected for fragmentation.

Peptide separation of samples measured on timsTOF Pro (Bruker Daltonics, Billerica, USA) was performed as previously described[12] on Bruker Daltonic's nanoElute LC system using an acclaim TM PepMap (Thermo Fisher Scientific) and a 75 μm x 25 cm Aurora Series emitter column (IonOpticks, Fitzroy, Australia). Peptides were separated along a gradient ranging from 0% to 95% Solvent B (ACN with 0.01% formic acid (FA)) over 60 min with consecutive ramps from 0% to 32% (30 min) and 32% to 40% (15 min), followed by two 5 min ramps to 60% and 95%, respectively. The peptides were subsequently analyzed in the online-coupled trapped ion mobility spectrometry and time-of-flight mass spectrometer timsTOF Pro equipped with a CaptiveSpray ion source using DDA implementing 6 parallel accumulation serial fragmentation (PASEF) ramps

with 1.44 s cycle time in combination with $1/k_0$ of 0.6-1.6 Vs/cm^2. Mass ranges were limited to 100-2,000 m/z, permitting charge states 2-5 (100-600 m/z) or charge states 1-5 (600-2,000 m/z).

Peptide separation of samples measured on the LTQ Orbitrap XL hybrid mass spectrometer (Thermo Fisher, Waltham, MA, USA) was performed by reversed-phase LC (nano-UHPLC, UltiMate 3000 RSLCnano; Thermo Fisher) and subsequently analyzed via MS. Samples were measured by injecting 5 µL per run onto a 75 µmx2 cm trapping column (Acclaim PepMap RSLC; Thermo Fisher) at 4 µl/min for 5.75 min. Peptide separation was subsequently performed at a flow rate of 175 nl/min on a 50 µm×50 cm separation column (Acclaim PepMap RSLC; Thermo Fisher) applying a gradient ranging from 2.4 to 32.0% of acetonitrile over 140 min. Eluted peptides were analyzed in the mass spectrometer implementing a top five CID (collision-induced dissociation) method generating fragment spectra for the five most abundant precursor ions in the survey scans with a resolution of 60,000, a mass range limited to 400-650 m/z for HLA class I peptides and 300-1,500 m/z for HLA class II peptides; positive charge states 2–3 for HLA class I and ≥ 2 for HLA class II were selected for fragmentation.

Web application

The PCI-DB is accessible *via* a web interface (https://pci-db.org/), which allows querying the database for peptide sequence and filtering by various parameters. It also enables manual spectrum inspection of any peptide in the database. The website provides multiple interactive overview visualizations implemented in JavaScript and D3.js. A PostgreSQL database stores the peptide data and all corresponding metadata. The back-end of the web application is implemented in Django, which connects to the PostgreSQL server using its Object-Relational Mapping (ORM) system. Gunicorn, a Python Web Server Gateway Interface (WSGI) HTTP server, hosts the web application server. Nginx is deployed as a reverse proxy. It performs the Secure Sockets Layer

(SSL) encryption and forwards requests to the Gunicorn server. Each component, PostgreSQL, Gunicorn Django server, and Nginx, is containerized using Docker and can be deployed together using Docker Compose.

Gene panel sequencing

The SureSelect HS Somatic Cancer Panel v4 was used to sequence tumor and normal samples. Tumor samples were compared against a corresponding donor blood ethylenediaminetetraacetic (EDTA) sample used as a normal reference. The tumor sample was sequenced at a coverage of 500x and the normal sample at 400x. Sequencing data was analyzed using the megSAP pipeline[13].

Personalized peptide vaccine production and application

Composition, production, and application of the personalized peptide vaccine within a compassionate use program (expanded access) for personalized peptide vaccination under the project clinicaltrials.gov NCT05014607 was performed as described previously[14]. In brief, the peptides were produced by the Good Manufacturing Practices (GMP) Peptide Laboratory of the Department of Immunology, University Tübingen, Germany, and combined with the adjuvant lipopeptide synthetic TLR1/2 ligand XS15[15] (manufactured by Bachem AG, Bubendorf, Switzerland) emulsified in Montanide™ ISA51 VG (manufactured by Seppic, Paris, France)[16]. The peptide vaccine was subcutaneously injected into the lower abdomen of the patients.

Patients and Blood Samples

Blood from cancer patients was collected at the University Hospital Tübingen as part of a personalized peptide vaccine treatment within a compassionate use program (expanded access) for personalized peptide vaccination under the project clinicaltrials.gov NCT05014607. The local Ethics Committee (406/2019BO2) approved the project, which was conducted under the German

Drug Law §13 paragraph 2b. All patients provided written informed consent prior to any measure. Peripheral blood mononuclear cells (PBMCs) were isolated by density gradient centrifugation, frozen in cryovials and stored at -80°C or in N_2 nitrogen until further use for subsequent T cell-based assays.

Amplification of peptide-specific T-cells and IFN-γ ELISpot Assay

PBMCs isolated from cancer patients within a compassionate use program were stimulated and analyzed as described previously[14]. An irrelevant peptide was used as negative control (ETVITVDTKAAGKGK or HLA class II (source protein: FLNA_HUMAN)). PBMCs isolated from cancer patients were stimulated with the vaccine peptides and the negative control peptide (5 µg/ml), cultured for 12 days with addition of 20 U/ml IL-2 (Novartis, Basel, Switzerland) on days 2, 5, 7 and optionally 9. Peptide-stimulated PBMCs were analyzed by IFN-γ ELISpot assay on day 12[17,18]. T-cell responses are evaluated by calculated spot counts, which are determined by subtracting the respective negative controls from the mean spot counts of the technical replicates. T-cell responses were considered positive if more than 10 spots per 500,000 cells were detected and the mean spot count was at least three-fold higher than the one in the negative control.

Intracellular Cytokine Staining (ICS)

The functionality of vaccine peptide-specific T cells was analyzed by intracellular cytokine staining, as previously described[18-20]. Cells were stained with APC/Cy7 anti-human CD4 (BioLegend, San Diego, CA, USA), PE-Cy7 anti-human CD8 (Beckman Coulter, Brea, CA, USA), APC anti-human IL-2 BioLegend, San Diego, CA, USA), FITC anti-human CD107a (BioLegend, San Diego, CA, USA), PE anti-human IFN-γ (BioLegend, San Diego, CA, USA), APC/Cy7 anti-human CD4 (BD BioSciencs, Franklin Lakes, New Jersey, USA), PE anti-human IL-2 (BioLegend, San Diego, CA, USA), BV421 anti-human IFN-γ (BioLegend, San Diego, CA,

USA), BV605 anti-human TNF (BioLegend, San Diego, CA, USA), FITC anti-human CD107a (BD BioSciencs, Franklin Lakes, New Jersey, USA), APC anti-human aCD154 (BioLegend, San Diego, CA, USA), as well as Zombie Aqua (BioLegend, San Diego, CA, USA). Dimethyl sulfoxide (DMSO) and a negative control peptide were used as negative controls. Samples were considered positive if the frequency of peptide-specific maker-positive T cells was ≥ 0.1 % of all viable single $CD4^+$ or $CD8^+$ cells and at least three-fold higher in the corresponding negative control. Samples were analyzed on FACS Fortessa/Canto II cytometers (BD, Franklin Lakes, NJ, USA). The gating strategies of the analysis are provided in Fig. S15.

Graphical abstract

The graphical abstract was created with BioRender[21].

Supplementary references

1. Bichmann L, Nelde A, Ghosh M, et al. MHCquant: automated and reproducible data analysis for immunopeptidomics. *Journal of proteome research* 2019;18(11):3876-84. doi: 10.1021/acs.jproteome.9b00313
2. Ewels PA, Peltzer A, Fillinger S, et al. The nf-core framework for community-curated bioinformatics pipelines. *Nature biotechnology* 2020;38(3):276-78. doi: 10.1038/s41587-020-0439-x
3. Scheid J, Lemke S, Hoenisch-Gravel N, et al. MHCquant2 refines immunopeptidomics tumor antigen discovery. *Research Square* 2024 doi: 10.21203/rs.3.rs-5560023/v1
4. Eng JK, Hoopmann MR, Jahan TA, et al. A deeper look into Comet—implementation and features. *Journal of the American Society for Mass Spectrometry* 2015;26(11):1865-74. doi: 10.1007/s13361-015-1179-x
5. The M, MacCoss MJ, Noble WS, et al. Fast and accurate protein false discovery rates on large-scale proteomics data sets with percolator 3.0. *Journal of the American Society for Mass Spectrometry* 2016;27:1719-27. doi: 10.1007/s13361-016-1460-7
6. Declercq A, Bouwmeester R, Hirschler A, et al. MS2Rescore: data-driven rescoring dramatically boosts immunopeptide identification rates. *Molecular & Cellular Proteomics* 2022;21(8) doi: 10.1016/j.mcpro.2022.100266
7. Degroeve S, Martens L. MS2PIP: a tool for MS/MS peak intensity prediction. *Bioinformatics* 2013;29(24):3199-203. doi: 10.1093/bioinformatics/btt544
8. Bouwmeester R, Gabriels R, Hulstaert N, et al. DeepLC can predict retention times for peptides that carry as-yet unseen modifications. *Nature methods* 2021;18(11):1363-69. doi: 10.1038/s41592-021-01301-5
9. Weinstein JN, Collisson EA, Mills GB, et al. The cancer genome atlas pan-cancer analysis project. *Nature genetics* 2013;45(10):1113-20. doi: 10.1038/ng.2764
10. Mohr C, Gabernet G, Peltzer A, et al. nf-core/epitopeprediction: v2.2.1 - WaldhaeuserOst Hotfix - 2023-03-16. *Zenodo* 2023 doi: 10.5281/zenodo.7744192
11. Reynisson B, Alvarez B, Paul S, et al. NetMHCpan-4.1 and NetMHCIIpan-4.0: improved predictions of MHC antigen presentation by concurrent motif deconvolution and integration of MS MHC eluted ligand data. *Nucleic acids research* 2020;48(W1):W449-W54. doi: 10.1093/nar/gkaa379
12. Hoenisch Gravel N, Nelde A, Bauer J, et al. TOFIMS mass spectrometry-based immunopeptidomics refines tumor antigen identification. *Nature Communications* 2023;14(1):7472. doi: 10.1038/s41467-023-42692-7
13. Sturm M, Admard J, Schütz L, et al. imgag/megSAP. *Zenodo* doi: 10.5281/zenodo.13744182
14. Bauer J, Köhler N, Maringer Y, et al. The oncogenic fusion protein DNAJB1-PRKACA can be specifically targeted by peptide-based immunotherapy in fibrolamellar hepatocellular carcinoma. *Nature Communications* 2022;13(1):6401. doi: 10.1038/s41467-022-33746-3
15. Rammensee H-G, Wiesmüller K-H, Chandran PA, et al. A new synthetic toll-like receptor 1/2 ligand is an efficient adjuvant for peptide vaccination in a human volunteer. *Journal for immunotherapy of cancer* 2019;7:1-18. doi: 10.1186/s40425-019-0796-5
16. Aucouturier J, Dupuis L, Deville S, et al. Montanide ISA 720 and 51: a new generation of water in oil emulsions as adjuvants for human vaccines. *Expert review of vaccines* 2002;1(1):111-18. doi: 10.1586/14760584.1.1.111
17. Kowalewski DJ, Schuster H, Backert L, et al. HLA ligandome analysis identifies the underlying specificities of spontaneous antileukemia immune responses in chronic

lymphocytic leukemia (CLL). *Proceedings of the National Academy of Sciences* 2015;112(2):E166-E75. doi: 10.1073/pnas.1416389112
18. Schuhmacher J, Kleemann L, Richardson JR, et al. Simultaneous identification of functional antigen-specific CD8+ and CD4+ cells after in vitro expansion using elongated peptides. *Cells* 2022;11(21):3451. doi: 10.3390/cells11213451
19. Nelde A, Maringer Y, Bilich T, et al. Immunopeptidomics-guided warehouse design for peptide-based immunotherapy in chronic lymphocytic leukemia. *Frontiers in immunology* 2021;12:705974. doi: 10.3389/fimmu.2021.705974
20. Schöllhorn A, Schuhmacher J, Besedovsky L, et al. Integrin activation enables sensitive detection of functional Cd4+ and Cd8+ T cells: Application to characterize sars-Cov-2 immunity. *Frontiers in immunology* 2021;12:626308. doi: 10.3389/fimmu.2021.626308
21. Walz JS. Created in BioRender. https://BioRender.com/d33v352. 2025
