# Supplementary figures and images for: PCI-DB: a novel primary tissue immunopeptidome database to guide next-generation peptide-based immunotherapy development
Source: J Immunother Cancer. 2025 Apr 15;13(4):e011366. doi: 10.1136/jitc-2024-011366 (PMC12001369; doi:10.1136/jitc-2024-011366)

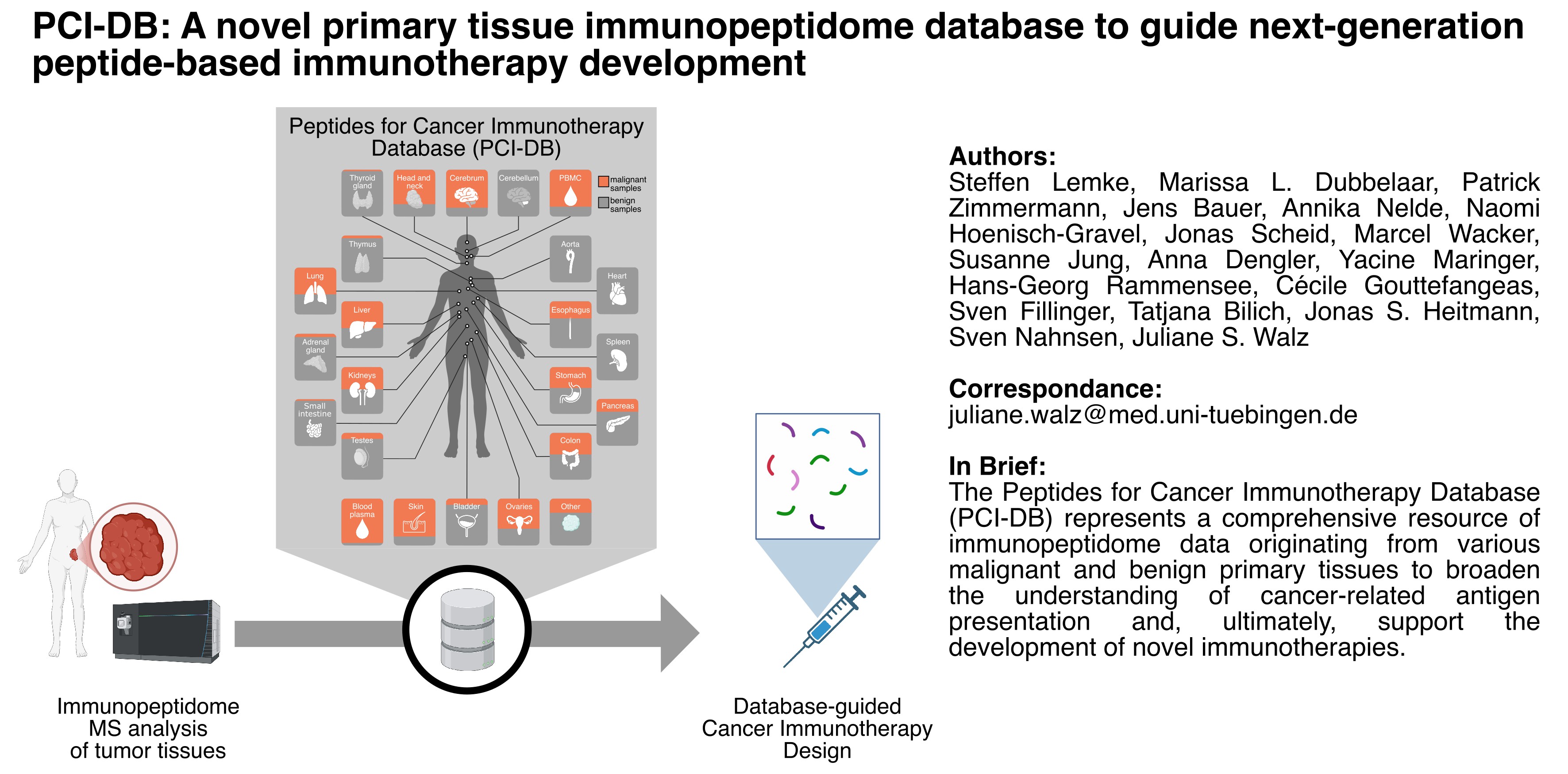

Supplement: online supplemental file 4 [file jitc-13-4-s005.jpg]
